# Multisectoral drivers of decarbonizing battery electric vehicles in China

**DOI:** 10.1093/pnasnexus/pgad123

**Published:** 2023-05-16

**Authors:** Fang Wang, Shaojun Zhang, Yinan Zhao, Yunxiao Ma, Yichen Zhang, Anders Hove, Ye Wu

**Affiliations:** School of Environment, State Key Joint Laboratory of Environment Simulation and Pollution Control, Tsinghua University, Beijing 100084, People’s Republic of China; School of Environment, State Key Joint Laboratory of Environment Simulation and Pollution Control, Tsinghua University, Beijing 100084, People’s Republic of China; State Environmental Protection Key Laboratory of Sources and Control of Air Pollution Complex, Beijing 100084, People’s Republic of China; Beijing Laboratory of Environmental Frontier Technologies, School of Environment, Tsinghua University, Beijing 100084, People’s Republic of China; School of Environment, State Key Joint Laboratory of Environment Simulation and Pollution Control, Tsinghua University, Beijing 100084, People’s Republic of China; School of Environment, State Key Joint Laboratory of Environment Simulation and Pollution Control, Tsinghua University, Beijing 100084, People’s Republic of China; School of Environment, State Key Joint Laboratory of Environment Simulation and Pollution Control, Tsinghua University, Beijing 100084, People’s Republic of China; GIZ and Research Associate, Oxford Institute for Energy Studies; School of Environment, State Key Joint Laboratory of Environment Simulation and Pollution Control, Tsinghua University, Beijing 100084, People’s Republic of China; State Environmental Protection Key Laboratory of Sources and Control of Air Pollution Complex, Beijing 100084, People’s Republic of China; Beijing Laboratory of Environmental Frontier Technologies, School of Environment, Tsinghua University, Beijing 100084, People’s Republic of China

**Keywords:** life-cycle assessment, electric vehicle, CO_2_ emissions, battery, automotive metals

## Abstract

China has made great progress in the electrification of passenger cars, and the sales of battery electric vehicles (BEVs) have exceeded 10%. We applied a life-cycle assessment (LCA) method to estimate the carbon dioxide (CO_2_) emissions of the past (2015), present (2020), and future (2030) BEVs, incorporating China's carbon peaking and neutrality policies, which would substantially reduce emissions from the electricity, operation efficiency, metallurgy, and battery manufacturing industries. BEVs can reduce cradle-to-grave (C2G) CO_2_ emissions by ∼40% compared with internal combustion engine vehicles (ICEVs) on the national-average level in 2020, far more significant than the benefit in 2015. Improved BEV operating efficiency was the largest factor driving emission reductions from 2015 to 2020. Looking forward to 2030, China's BEVs equipped with nickel–cobalt–manganese (NCM) batteries can achieve a further 43% of CO_2_ emissions reductions, among which 51 g km^−1^ of reduction is from the well-to-wheels (WTW) stage majorly owing to the further cleaner electricity mix, while other vehicle-cycle benefits are mainly from the advancement of battery (12 g km^−1^) and related metal materials (5 g km^−1^). We highlight the importance of better material efficiency and synchronized decarbonization through the automotive industrial chain in promoting climate mitigation from transport activities.

**Significance Statement**

Multiple complex sectors can greatly affect the life-cycle carbon dioxide (CO_2_) emissions of battery electric vehicles (BEVs). Based on real-world-featured investigation and near-term projection data across multiple industries, including electricity, vehicle fuel economy, major automotive metals, and battery, we dissect the multiplesectoral impacts on decarbonizing cradle-to-grave CO_2_ emissions for BEVs in China. The results will be of significant value on the design of incentive policies for BEVs, a landmark type of end-use sectors in this energy-transition era, to coordinate the decarbonization associated with important upstream industries.

## Introduction

As the end-use sector with the highest reliance on fossil fuels, the global transport sector (8.3 Gt) was responsible for ∼23% of total combustion carbon dioxide (CO_2_) emissions before the Covid-19 pandemic (1). Among all transport modes, light-duty vehicles, predominantly passenger cars, were the largest contributor to the total transport CO_2_ emissions (1). Electrifying passenger cars are regarded as one predominant solution to deliver deep mitigation of their CO_2_ emissions in the future. By the end of 2021, there have been 16.5 million passenger electric vehicles [EVs; including 11.3 million battery electric vehicles (BEVs)] throughout the world (2). As the largest BEV market since 2016, China contributed 51% of global EV sales in 2021 (2) and will continue the ambition of accelerating the electrification trend in the future (3). The updated New Energy Vehicle (NEV) Industrial Development Plan (2021–2035) has announced a near-term target of increasing the sales share of NEVs to 20% by 2025 (13.4% by 2021) (4). It is further estimated that BEVs will account for nearly 30 to 40% of the total sales of passenger cars by 2030 (5).

The potential environmental and climate benefits of BEVs provide an important motivation for China's supportive policies in developing BEVs (6). However, considering the high reliance on coal-based electricity generation, there remains ongoing public debate on the actual impacts of BEVs on mitigating CO_2_ emissions. Life-cycle assessment (LCA) methods then were developed to address this issue, which could be typically categorized into two types according to the boundary setting, well-to-wheels (WTW, only focusing on the fuel cycle) or cradle-to-grave (C2G, further including production and recycling processes of automotive components materials). The early-stage LCA studies focused on the WTW research since the fuel cycle then was estimated to be the major proportion of total life-cycle CO_2_ emissions (i.e. C2G). Huo et al. (7) and Wu et al. (8) similarly concluded that around 2010, BEVs had very limited benefits in mitigating WTW CO_2_ emissions relative to gasoline cars in coal power-rich regions (e.g. North China), whereas BEVs could effectively mitigate WTW CO_2_ emissions in South China, given its relatively cleaner electricity mix. Several follow-up studies regarding WTW emissions further enriched the analysis of the impact by considering the spatial or temporal divergence of the electricity mix (9–18). These studies have reported the decline of WTW CO_2_ emissions for BEVs in China due to the cleaner electricity mix, and BEVs could readily deliver WTW CO_2_ mitigation even in North China. Few recent studies have attempted to introduce localized profiles regarding some elementary vehicle-cycle components into the C2G boundary level (19–23). These studies have also confirmed the reductions in C2G CO_2_ emissions for BEVs in China, but note a considerable share [e.g. 25% estimated by the International Council on Clean Transportation (ICCT) (22)] contributed by the vehicle cycle.

It is worth noting that multiple complex factors apart from the electricity mix can affect the C2G emissions of BEVs. In contrast to the early stage of initial market adoption, the energy density of automotive lithium-ion (Li-ion) batteries and electricity consumption of BEVs have improved substantially in the past few years. Thus, we emphasize that BEVs and internal combustion engine vehicles (ICEVs) at fleet level should be selected as compared components, whose key characteristics (e.g. vehicle weight, battery performance, and fuel economy) represent China's sales-weighted trends of vehicle markets. On the other hand, mature automotive metal industries (e.g. steel, aluminum, and copper) have made limited contributions to decarbonizing, and their net-zero transition pathways are not clear meanwhile, though China's plan for achieving a carbon peak before 2030 and carbon neutrality by 2060 will ultimately affect these industries. Regarding these multisectoral drivers, there have been limited efforts in the analysis and synthesis of cross-sector impacts on decarbonizing BEVs. This study has comprehensively dissected the cross-sector drivers. We analyzed the WTW CO_2_ for BEVs reflecting the change brought by electricity consumption and average electricity mix. Next, CO_2_ emissions generated in major metal production and battery production were estimated with investigated data. Furthermore, we synthesized all the drivers to evaluate the potential C2G CO_2_ emissions reduction in 2030 with each factors dissected. Results can help improve the design of low-carbon policies for automotive manufacturers and associated electricity, metallurgy, and battery manufacturing industries.

## Results

### WTW CO_2_ emissions for BEVs across all subgrid regions

**Fig. 1.**
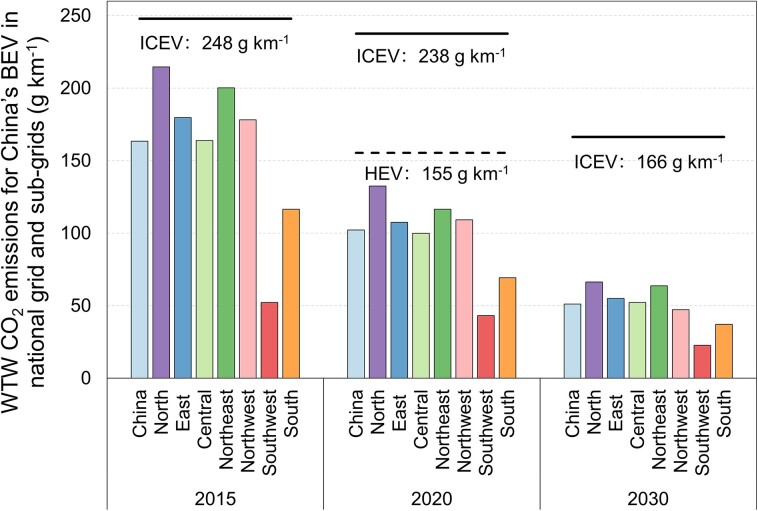
WTW CO_2_ emissions for China's BEVs in national grid and subgrids (2015, 2020, and 2030).

BEVs are estimated to mitigate WTW CO_2_ emissions by 57% in 2020 with the national-average grid [102 g km^−1^ for BEVs, 155 g km^−1^ for hybrid electric vehicles (HEVs), and 238 g km^−1^ for ICEVs] and could deliver WTW CO_2_ emission mitigations across all subgrid regions (see Fig. 1). In the most coal-dependent region, the North subgrid (a coal-based share of 79%), BEVs could still reduce 44% WTW CO_2_ emissions compared with the ICEV counterparts there. In cleaner subgrid regions, such as the South and Southwest, the mitigation percentages could be enlarged to 71 and 82%. The national-average reduction of WTW CO_2_ emissions for BEVs than ICEVs (57%) is higher than the estimates for previous years [e.g. 20% in 2010 by Huo et al. (9), 31% in 2017 by Gan et al. (14), and 36% in 2020 by Ehrenberger (17)]. Alongside the cleaner electricity mix with year, the discrepancies can be majorly attributed to the lower electricity consumption of BEVs in this study compared with previous studies [e.g. 15 kWh 100 km^−1^ in 2020 in this study vs. 23 kWh 100 km^−1^ in Gan et al. (14)]. For HEVs, their WTW CO_2_ could be reduced by 35% compared with conventional ICEVs due to the on-road oil-saving benefits.

The advantages of BEVs back in 2015 were not as substantial as those in the present. BEVs could only reduce WTW CO_2_ emissions by 34% with the national-average electricity mix, and the reduction was much less in North China (13%) and Northeast China (19%). In addition to the progress brought by the penetration of renewable power and the improved generation efficiency of thermal power, another important cause is that BEVs have improved their real-world fuel economy much more effectively than ICEVs over the years. A detailed analysis of the drivers of decarbonizing BEVs in this historical period will be presented in a later section.

Looking forward to 2030, BEVs will depict stronger efficacy in mitigating WTW CO_2_ emissions even if a sufficient improvement in the fuel economy of ICEVs has been assumed to reflect the possible widespread HEVs. The national-average WTW CO_2_ emissions of BEVs are estimated to decrease to nearly 50 g km^−1^ in 2030, ∼70% lower than that of ICEVs then. Among all subgrid regions, the Northwest and North subgrids will embrace rapid installations of renewable electricity in the coming decade, which will accelerate the decline of WTW CO_2_ emissions for BEVs there. For example, WTW CO_2_ emissions are estimated to be about 47 g km^−1^ in the Northwest subgrid, lower by 71% than those of ICEVs.

### Life-cycle CO_2_ emissions of producing major automotive metals

**Fig. 2.**
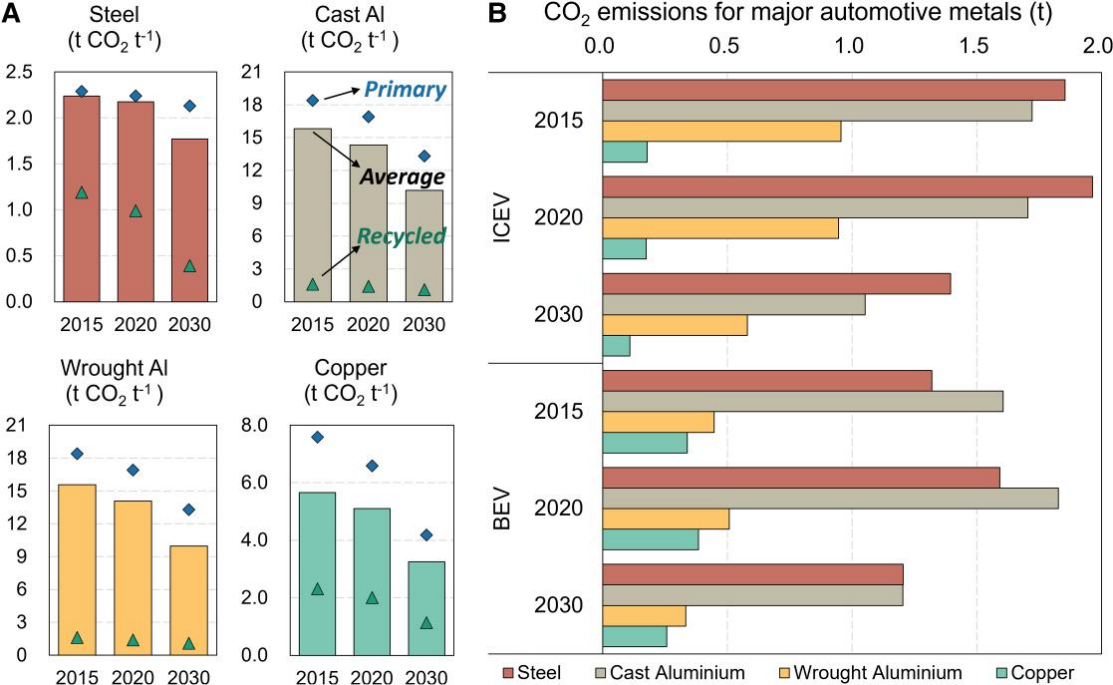
CO_2_ emission intensities for major metals and their contribution to a passenger car. Panel a) indicates the average CO_2_ emission intensity of major metal production as well as the CO_2_ emission intensity of primary and recycled metal production. Panel b) estimates the CO_2_ emissions associated with the production of major metals per passenger vehicle.

The estimated average CO_2_ emission intensities per ton of steel, cast aluminum, wrought aluminum, and copper are 2.2, 14.3, 14.1, and 5.1 t in 2020, respectively (see Fig. 2a). These results, as vital basis for 2030 estimation, show good consistency with the previous studies (24–26). Compared with 2015, very limited progress has been achieved due to the lack of policy pressure. Now that China has set a firm target for achieving the carbon peak before 2030, the metal industries face tremendous pressure to promote decarbonization. Our results indicate that 19% for steel and nearly 30% for others of CO_2_ emission mitigation will be achievable by 2030 with the increased use of recycled materials and advanced metal smelting technologies. The total life-cycle CO_2_ emissions of these major metallic materials used in ICEVs and BEVs in 2020 are 4.8 and 4.3 t veh^−1^ and are expected to decrease by 30 to 35% in 2030 (see Fig. 2b).

The smelting of aluminum and copper heavily relies on electricity. Our analysis suggests that promoting recycled materials has played a useful role in decarbonizing metal production. Taking cast aluminum for example, the CO_2_ emission intensity of producing primary materials in 2020 is 16.9 t, while utilizing recycled materials is only 1.4 t, ∼8.5% of the primary ones. The blending rate of 19% recycled aluminum in 2020 helped the average emissions intensity reduced by 15% from primary materials. If considering only the improvement of primary materials technology, the reduction is no higher than 8% since 2015 though the electricity consumption and the carbon intensity of electricity both decreased, from where the significant benefits of recycling can be seen. In the future, with the phaseout of captive coal-fired power plants for aluminum industries (63% in 2020 vs. 44% in 2030), the life-cycle CO_2_ emission intensity of primary cast aluminum is expected to decrease to 13.3 t in 2030, and a blending rate of 26% of recycled aluminum can further reduce the CO_2_ emission intensity by 24%. Similar trends are also found for wrought aluminum. For copper, the life-cycle CO_2_ emissions intensities are estimated to be 2.0 and 6.6 t in 2020, respectively, for recycled and primary copper. The utilization rate of recycled copper was 32% in 2020 and is expected to relatively stabilize till 2030, for the demand for total copper and supply of recycled copper are expanding at a similar pace. At that moment, the average CO_2_ emission intensity of copper production will be 3.3 t (5.1 t in 2020) mainly due to the improvement of electricity carbon intensity.

Different from the above two electricity-intensive metals, steel smelting is highly dependent on coal (used as reductant and process fuel) and therefore faces a higher degree of difficulty in mitigating CO_2_ emissions. The average CO_2_ emission intensity of primary steel in 2020 only decreased by 2% since 2015. Under this situation that new technologies both low cost and low carbon are not clear so far, the surest decarbonizing way is to promote the usage of recycled steel. High-carbon blast furnace–basic oxygen furnace (BF-BOF, 94.9% of total capacity) and low-carbon scrap-based electric arc furnace (EAF, 5.1% of total capacity) are two major technologies for steel smelting in 2020, and both technologies can use scrap steel as raw materials. The penetration of scrap steel was 12 and 51% for BF-BOF and EAF, and the CO_2_ emission intensities were 2.2 and 0.99 t, respectively. In 2030, with the increase of EAF capacity (15%) and the increase of scrap steel penetration in EAF (100%), the CO_2_ emission intensity of the EAF pathway is expected to achieve 0.39 t, and the overall average life-cycle CO_2_ emission intensity of steel will be 1.8 t.

### Life-cycle CO_2_ emissions of NCM and LFP batteries

**Fig. 3.**
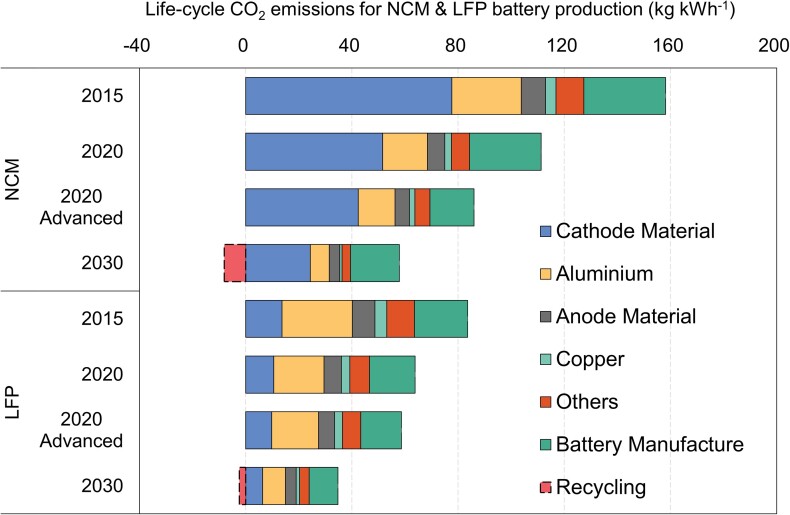
Average life-cycle CO_2_ emissions for NCM and LFP batteries (2015, 2020, and 2030). The error bars indicate the emission variations associated with the battery manufacture process according to the investigation data of five plants. The 2020 advanced levels of NCM and LFP are estimated with the advanced energy density (180 Wh kg^−1^ for NCM and 140 Wh kg^−1^ for LFP, both at the pack level) and manufacture-related CO_2_ emissions data.

Fig. 3 presents the average life-cycle CO_2_ emissions of nickel–cobalt–manganese oxide (NCM) and lithium iron phosphate (LFP) battery packs by three processes (production of raw materials, manufacture of battery from cell to pack, and battery recycling), and four important raw materials (cathode, anode, aluminum, and copper) are further distinguished. In 2020, the average life-cycle CO_2_ emissions for producing NCM and LFP batteries are estimated to be 111 and 64 kg kWh^−1^, respectively, which are equivalent to typical ranges given by a review article (27). For both battery types, the production of raw materials is the leading contributor (76 and 73%) in total life-cycle CO_2_ emissions, of which productions of cathode material and aluminum are carbon-intensive processes that together contribute to 82% for NCM and 63% for LFP in the total raw material-related CO_2_ emissions. The manufacturing of battery packs accounted for 24 and 27% of total life-cycle CO_2_ emissions for both NCM and LFP battery packs, respectively. It is noteworthy that advanced NCM and LFP batteries that have been commercially used in BEVs can achieve 86 and 59 kg kWh^−1^ of life-cycle CO_2_ emissions currently, whereas the battery manufacture process denotes 17 and 15 kg kWh^−1^. This result reflecting battery performance advancements was rarely reported in previous studies (27).

The life-cycle CO_2_ emissions are estimated to decrease by around 30% from 2015 to 2020, and the progress resulted from the improved energy density of the battery pack and the cleaner mix of electricity. Battery energy density as the leading factor has been increased by 41% during this period, which indicates that the material consumption per kWh battery could be reduced from 9.7 to 6.9 kg and the CO_2_ emissions could be reduced by 29% theoretically. For example, the production of NCM cathode materials could cause 78 kg CO_2_ kWh^−1^ in 2015 and 52 g/kWh in 2020. This 34% reduction resulted mainly from improved battery energy density, supplemented by a cleaner electricity mix for battery production. In the case of aluminum being used to produce cathode aluminum foil and heat dissipation aluminum plates, CO_2_ emissions intensity also dropped by 36% from 26 to 17 kg kWh^−1^. Similar battery cost reduction brought by energy density improvement was also revealed in a previous study (28).

In the future, we estimate that NCM and LFP battery packs will have lower life-cycle CO_2_ emissions (note: without battery recycling) of 58 and 35 kg kWh^−1^ in 2030, representing reductions of 47%. Among different phases, the proportion of battery pack manufacturing will be of greater significance. For NCM, the emission share of battery pack manufacturing in the total life cycle would increase to 32% in 2030, while that for LFP will remain quite stable (31% in 2030). It is worth noting that battery recycling will also relieve CO_2_ emissions by 8.0 and 2.4 kg kWh^−1^, as fewer new raw materials would be required. Further estimates could follow up to fulfill this issue as reusing retired batteries might have complicated scenarios and in particular technological challenges for NCM batteries.

### Dissecting the major drivers of decarbonizing CO_2_ emissions for BEVs in China

**Fig. 4.**
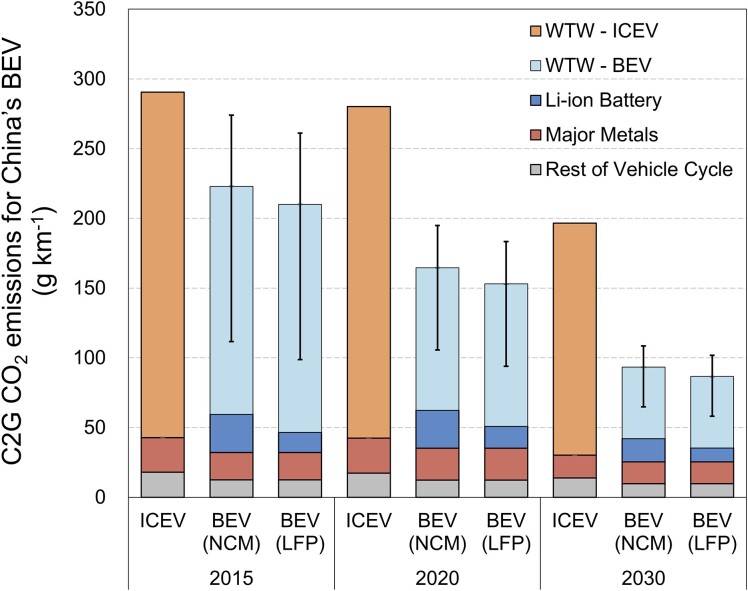
C2G CO_2_ emissions of ICEV and BEV during 2015–2030 with the national-average electricity mixes. The error bars represent the variability of WTW CO_2_ emissions due to the different electricity mixes across various subgrids.

The estimated C2G CO_2_ emissions of NCM-BEVs, LFP-BEVs, and ICEVs in 2020 are 165, 153, and 280 g km^−1^, respectively, as shown in Fig. 4. According to the national-average electricity mix, BEVs have already shown a ∼40% reduction in C2G CO_2_ emissions compared with ICEVs in 2020 (sensitivity analysis is available in Fig. S9) and the reduction would further increase to 53% in 2030, despite only a 23% reduction in 2015. As far as subgrid regions are concerned, the C2G CO_2_ mitigations of NCM-BEVs range between 30 and 62%. This indicates that even in the North grid still with a high proportion of coal-fired electricity, BEVs can achieve considerable emission reduction benefits. Since lifespan is a vital parameter to allocate WTW and vehicle-cycle emissions, we have tested that NCM-BEVs could still have C2G emission mitigation in the North subgrid relative to ICEVs if the lifespan is greater than 100,000 km. Thus, we conclude that the life-cycle CO_2_ mitigation benefits of promoting BEVs in China are fully demonstrated.

Another noteworthy point is that the vehicle cycle has contributed to 38% of total C2G CO_2_ emissions for NCM-BEVs, and this share of LFP-BEVs is also above 30%. For NCM-BEVs, the productions of battery and major metal materials (steel, aluminum, and copper) share the majority of vehicle-cycle CO_2_ emissions, accounting for 16 and 14%, respectively, of the C2G CO_2_ emissions. The proportion of vehicle-cycle CO_2_ emissions is higher than that in previous studies in China due to our mitigated estimation of WTW emission burden [e.g. an average of 29% among 23 to 60% by Wu et al. (21) and 24% by the ICCT (22)], though the emission intensity in Wu et al. (21) (62 g km^−1^) is the same as our estimation. Meanwhile, this number is also higher than 31% (43 g km^−1^) in the United States, according to the GREET model, while the main difference comes from their larger lifespan to be allocated. In 2030, we estimate the proportion would further increase to 45% in China. Therefore, decarbonizing BEVs could not merely rely on the progress of the power sector but should engage all relevant supply chains in particular battery and major metal industries (29).

**Fig. 5.**
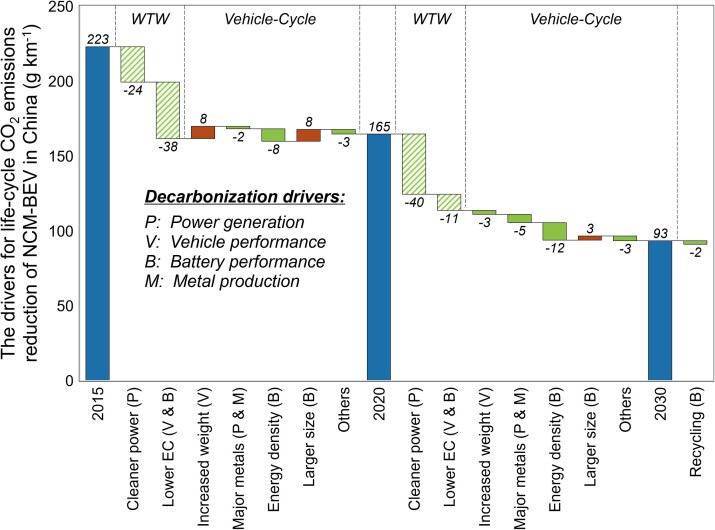
Drivers on decarbonizing NCM-BEVs from 2015 to 2030. The drivers are categorized into two phases (WTW and vehicle cycle) and four major parts: P, power generation; V, vehicle performance [e.g. vehicle weight, energy consumption (EC)]; B, battery performance (e.g. battery size and energy density); and M, metal production. See Fig. S1 for the results of LFP-BEVs.

Multiple factors combined to deliver the rapid decarbonization of C2G CO_2_ emissions for BEVs during 2015–2020. Taking NCM-BEVs for example, Fig. 5 clearly illustrates that improved efficiency of BEVs, a factor that studies have often omitted, was more important than the cleanness of the electricity mix (see Fig. S1 for LFP-BEVs). In the WTW stage with other factors unchanged, cleaner power alone can reduce 24 g CO_2_ km^−1^; on this basis, the improvement of efficiency can lead to a reduction of 38 g CO_2_ km^−1^. Therefore, the WTW CO_2_ emissions can be reduced by 28% owing to advancements in electricity, vehicle technology, and charging efficiency. In the vehicle cycle, the benefit from the improved energy density is offset by the increased battery capacity, indicating that improved battery technology could largely avoid higher CO_2_ emissions due to the higher all-electric range (AER) that attracts more BEV consumers. However, the increased vehicle weight was a negative factor to increase C2G CO_2_ emissions. For LFP-BEVs (see Fig. S1), the characteristics of these decarbonization drivers are proportionally comparable to NCM-BEVs.

With the accelerated decarbonization of the power system during 2020–2030, we estimate that the future cleanness of the electricity mix will be the major driver to reduce C2G CO_2_ emissions for BEVs. The total reductions then will reach 43% (72 and 66 g km^−1^) for the two types of BEVs. For NCM-BEVs, the cleaner electricity will lead to 40 g km^−1^ of C2G CO_2_ emission mitigation. The improved operating and charging efficiency would be other drivers to reduce WTW CO_2_ emissions by 11 g km^−1^ (thus jointly reducing the WTW CO_2_ emissions by 51 g km^−1^). The improvement in the energy density of the battery is still significant, resulting in 12 g km^−1^ of C2G CO_2_ mitigation. The decarbonized production of metal materials can also contribute to emission mitigation by 5 g km^−1^. Battery recycling can bring additional emissions mitigation of 2 g km^−1^. Continued improvements in BEV efficiency will bring declining marginal benefits relative to 2015–2020. Even if the official operating efficiency improved to 10 kWh per 100 km, the C2G CO_2_ emission mitigation would only improve by 4 g km^−1^.

## Discussion

The Council of the European Union (EU) adopted a proposal for strengthening the regulation on batteries to ensure more sustainable use of batteries along the entire value chain (30). A carbon footprint limit will be introduced for EV batteries, an important component accounting for a considerable fraction of the total C2G CO_2_ emissions of BEVs as identified in this study. The new EU battery regulation proposal highlights a trend that LCA will play a more vital role in future climate mitigation management. Due to the limited availability of original industry-based data, many existing LCA studies on life-cycle CO_2_ emissions of automotive batteries and BEVs heavily relied on secondary information from other publications. We obtained primary data from multiple plants through the value chains of battery and automotive industries in China, which can improve the reliability and robustness of industry-average carbon footprints for better decision-making by industrial and governmental stakeholders. Furthermore, this study fully updates LCA estimates with real-world data regarding vehicle specifications in China, incorporates realistic forecasts for future emissions reductions in the power and metals sectors due to China's 2030 and 2060 carbon peaking and carbon neutrality policies, and utilizes these factors to analyze the major drivers of decarbonizing passenger vehicle transport emissions. This can inform policymakers of a clear understanding of the complicated tasks to reduce C2G emissions of BEVs during the electrification megatrend, in particular by clarifying the necessity of improving vehicle and battery technologies and decarbonizing automotive supply chains (e.g. steel, aluminum, and copper). Setting life-cycle CO_2_ limits for BEVs, an example of important high-value end-user products, can create opportunities to leverage and synchronize the decarbonization across many relevant industries.

To limit global warming below 1.5°C, CO_2_ emissions from combustion and production of fuels and electricity for transportation need to decline by at least 80% in 2050 compared with the current levels by 2050 (22). The challenging mission is feasible for China if a 36% reduction could be achieved by 2035 (3). Here, we have demonstrated that BEVs could readily reduce C2G CO_2_ emissions by more than a half in 2030 compared with ICEVs. Rapidly electrifying the passenger vehicle fleet should be an important policy orientation in China to comply with the climate target.

We acknowledge that there might be additional drivers to further increase the CO_2_ mitigation benefits for BEVs, which could be updated in the future. First, adopting average electricity mix during 2030 to 2040 for newly registered cars in 2030 or marginal electricity mix that associated with smart charging coordination and vehicle-to-grid technologies could further reduce WTW CO_2_ emissions for BEVs by accommodating more renewable energy (31–34). However, these advanced charging schemes have not been commercially adopted yet in China. Second, unlike many prior studies often comparing BEVs with ICEVs by vehicle segment, we abstracted China's whole BEV and ICEV fleet into two opposite vehicle models to reflect their respective trends in actual fleet-level characteristics. This approach may attribute uncertainties if BEVs penetrate more into larger segments in the future (see Table S11). Third, detailed life-cycle inventories (LCI) data of Li-ion batteries and their key components should be improved as more investigation data become available. Reusing Li-ion batteries can be incorporated into the recycling phase to potentially reduce life-cycle CO_2_ emissions. Finally, more progressive smelting technologies plus carbon capture and relocation of industry capacity aiming to shift captive coal-fired power to renewable electricity can achieve deep CO_2_ reductions in the future. As we do not specifically consider plug-in hybrid electric vehicles (PHEVs) in this study, we expect that the decarbonization efforts will also deliver life-cycle carbon mitigation benefits for PHEVs. All these untapped factors have the potential of delivering further CO_2_ mitigation and could be synchronized through the supply-chain low-carbon management of BEVs in the future policy framework.

## Materials and methods

### Scope and model description

We selected model years (MY) of 2015, 2020, and 2030 as the past, present, and future study years to compare C2G CO_2_ emissions between BEVs and ICEVs. It should be noted that China's annual sales of BEVs (including cars and other commercial vehicles) increased from 0.25 million to 2.92 million from 2015 to 2021, adequately representing the early stage of the widespread adoption of BEVs. C2G CO_2_ emissions are expressed in the CO_2_ emissions per unit distance traveled during the vehicle lifespan. The LCA study consists of two main aspects (see the LCA scope in Fig. S2): the fuel cycle (i.e. WTW) and the vehicle cycle and thus the C2G emissions are calculated as Eq (1).


(1) $$E_{C2G}=\frac{\mathrm{FC}\times EF_{\mathrm{WTW}}÷100}{{\mathrm{Eff}}_{\mathrm{charging}}}+\frac{E_{\mathrm{major}\;\mathrm{metals}}+E_{\mathrm{battery}}+E_{\mathrm{others}}}{\mathrm{Lifespan}}$$

where *FC* refers to fuel consumption for ICEV and BEV in the unit of L 100 km^−1^ and kWh 100 km^−1^, respectively. EF_WTW_ is the CO_2_ emission factor for gasoline (g L^−1^) or electricity (g kWh^−1^), and Eff_charging_ represents the charging efficiency of chargers, which depicts the proportion available for batteries from the output of chargers (80% in 2015, 86% in 2020, and 92% in 2030).

The vehicle cycle in this paper, including the production, assembly, disposal, and recycling of vehicle materials, has a special focus on updating China's LCI for major vehicle metals, battery systems, and other vehicle materials or processes (e.g. fluids). *E*_major metals_, *E*_battery_, *E*_others_ are then used as records of their CO_2_ emissions. Taking the battery system for example, the major processes include the production of raw materials, manufacture of electrodes and other battery components, assembly of battery packages, recycling of end-of-life batteries. Lifespan refers to the total mileage in a vehicle lifespan [190,000 km in this study (15)] since the total vehicle-cycle emission are expressed in g km^−1^.

### Key parameters for WTW stage and vehicle cycle

#### WTW stage

Key parameters consist of the *FC* for ICEVs and BEVs and *EF* for conventional gasoline and electricity. *FC* for ICEVs and BEVs is estimated based on official average fuel/electricity consumption and previous research on the gap between real-world values and official ones (see Table S1). The CO_2_ emission intensity for gasoline is mainly derived from the greenhouse gases, regulated emissions, and energy use in transportation (GREET) model with localized information (15) (see Table S2). Regarding electricity as shown in Fig. S3, consumption-based regional electricity mixes and corresponding CO_2_ emission intensities are estimated for seven interconnected but relatively independent subgrids: North, East, Central, Northeast, Northwest, Southwest, and South (details can be found in Tables S3 and S4).

#### Vehicle cycle

Key parameters consist of the curb weight (see Table S5) and life-cycle CO_2_ emission intensities for major metals, batteries manufacture, and recycling. The vehicle-relevant information was mainly estimated based on the “Catalogue of Models of New-Energy Automobiles Exempt from Vehicle Acquisition Tax,” while CO_2_ emission intensities were determined by field investigation and further confirmation with statistics from industry associations. Specifically for crude metals production (see Figs. S4–S6 and Table S6 for detailed introductions), seven steel plants, 70 electrolytic aluminum plants, and three copper smelters across the past years have been investigated. Toward future, the progress of current technology and development of recycled materials were considered. Regarding battery production, totally five plants for two types of batteries (NCM and LFP) were investigated (data are available in Figs. S7 and S8 and Tables S7–S10). By 2030, recycling Li-ion batteries via hydrometallurgical technology will predominate, and the CO_2_ emission intensity was also considered.

## Supplementary Material

Supplementary material is available at *PNAS Nexus* online.

## Supplementary Material

pgad123_Supplementary_Data

## Data Availability

All data are included in the manuscript and/or supporting information.

## References

[1] International Energy Agency (IEA) . Data from “Net zero by 2050”. https://www.iea.org/reports/net-zero-by-2050.

[2] International Energy Agency (IEA) . Data from “Global EV outlook 2022”. https://www.iea.org/reports/global-ev-outlook-2022.

[3] International Council on Clean Transportation (ICCT) . Data from “Opportunities and pathways to decarbonize China's transportation sector during the fourteenth five-year plan period and beyond”.

[4] International Council on Clean Transportation (ICCT) . Data from “China's New Energy Vehicle Industrial Development Plan for 2021 to 2035”.

[5] China Society of Automotive Engineers (China-SAE) . Data from “Technology roadmap for intelligent & connected vehicles 2.0” (in Chinese).

[6] Liang X , et al 2019. Air quality and health benefits from fleet electrification in China. Nat Sustain. 2:962–971.

[7] Huo H , Zhang Q, Wang MQ, Streets DG, He K. 2010. Environmental implication of electric vehicles in China. Environ Sci Technol. 44:4856–4861. 20496930 10.1021/es100520c

[8] Wu Y , et al 2012. Energy consumption and CO_2_ emission impacts of vehicle electrification in three developed regions of China. Energy Policy 48:537–550.

[9] Huo H , Zhang Q, Liu F, He K. 2013. Climate and environmental effects of electric vehicles versus compressed natural gas vehicles in China: a life-cycle analysis at provincial level. Environ Sci Technol. 47:1711–1718. 23276251 10.1021/es303352x

[10] Ke W , Zhang S, He X, Wu Y, Hao J. 2017. Well-to-wheels energy consumption and emissions of electric vehicles: mid-term implications from real-world features and air pollution control progress. Appl Energy. 188:367–377.

[11] Shen W , Han W, Wallington TJ, Winkler SL. 2019. China Electricity generation greenhouse gas emission intensity in 2030: implications for electric vehicles. Environ Sci Technol. 53:6063–6072. 31021614 10.1021/acs.est.8b05264

[12] Zhao F , Liu F, Liu Z, Hao H. 2019. The correlated impacts of fuel consumption improvements and vehicle electrification on vehicle greenhouse gas emissions in China. J Clean Prod. 207:702–716.

[13] Zheng Y , et al 2020. Well-to-wheels greenhouse gas and air pollutant emissions from battery electric vehicles in China. Mitig Adapt Strat Glob Change. 25:355–370.

[14] Gan Y , et al 2021. Provincial greenhouse gas emissions of gasoline and plug-in electric vehicles in China: comparison from the consumption-based electricity perspective. Environ Sci Technol. 55:6944–6956. 33945267 10.1021/acs.est.0c08217

[15] Märtz A , Plötz P, Jochem P. 2021. Global perspective on CO_2_ emissions of electric vehicles. Environ Res Lett. 16:054043.

[16] Sacchi R , Bauer C, Cox B, Mutel C. 2022. When, where and how can the electrification of passenger cars reduce greenhouse gas emissions? Renew Sustain Energy Rev. 162:112475.

[17] Ehrenberger SI , Dunn JB, Jungmeier G, Wang H. 2019. An international dialogue about electric vehicle deployment to bring energy and greenhouse gas benefits through 2030 on a well-to-wheels basis. Transp Res Part D: Transp Environ. 74:245–254.

[18] Wu Z , et al 2018. Life cycle greenhouse gas emission reduction potential of battery electric vehicle. J Clean Prod. 190:462–470.

[19] Hao H , Qiao Q, Liu Z, Zhao F. 2017. Impact of recycling on energy consumption and greenhouse gas emissions from electric vehicle production: the China 2025 case. Resour Conserv Recycling. 122:114–125.

[20] He X , et al 2019. Economic and climate benefits of electric vehicles in China, the United States, and Germany. Environ Sci Technol. 53:11013–11022. 31415163 10.1021/acs.est.9b00531

[21] Wu Z , et al 2019. Assessing electric vehicle policy with region-specific carbon footprints. Appl Energy. 256:113923.

[22] International Council on Clean Transportation (ICCT) . Data from “A global comparison of the life-cycle greenhouse gas emissions of combustion engine and electric passenger cars”.

[23] Hsieh IL , et al 2022. An integrated assessment of emissions, air quality, and public health impacts of China's transition to electric vehicles. Environ Sci Technol. 56(11):6836–6846. 10.1021/acs.est.1c06148. 35171556

[24] Ma X , et al 2018. Life cycle assessment and water footprint evaluation of crude steel production: a case study in China. J Environ Manage. 224:10–18. 30025260 10.1016/j.jenvman.2018.07.027

[25] International Aluminium Institute (IAI) . Data from “Aluminium sector greenhouse gas pathways to 2050”.

[26] Hong J , et al 2018. Life cycle assessment of copper production: a case study in China. Int J Life Cycle Assess. 23:1814–1824.

[27] Arshad F , et al 2022. Life cycle assessment of lithium-ion batteries: a critical review. Resour Conserv Recycling. 180:106164.

[28] Ziegler MS , Song J, Trancik JE. 2021. Determinants of lithium-ion battery technology cost decline. Energ Environ Sci. 14:6074–6098.

[29] Ohno H , Nuss P, Chen W, Graedel TE. 2016. Deriving the metal and alloy networks of modern technology. Environ Sci Technol. 50:4082–4090. 26927531 10.1021/acs.est.5b05093

[30] Council of the European Union . Data from “Sustainable batteries: member states ready to start negotiations with parliament.” https://www.consilium.europa.eu/en/press/press-releases/2022/03/17/sustainable-batteries-member-states-ready-to-start-negotiations-with-parliament/.

[31] Gai Y , Wang A, Pereira L, Hatzopoulou M, Posen ID. 2019. Marginal greenhouse gas emissions of Ontario's electricity system and the implications of electric vehicle charging. Environ Sci Technol. 53:7903–7912. 31244061 10.1021/acs.est.9b01519

[32] Chen J , et al 2022. Emission mitigation potential from coordinated charging schemes for future private electric vehicles. Appl Energy. 308:118385.

[33] Xu L , Yilmaz HÜ, Wang Z, Poganietz W, Jochem P. 2020. Greenhouse gas emissions of electric vehicles in Europe considering different charging strategies. Transp Res Part D Transp Environ. 87:102534.

[34] Woody M , Vaishnav P, Craig MT, Lewis GM, Keoleian GA. 2021. Charging strategies to minimize greenhouse gas emissions of electrified delivery vehicles. Environ Sci Technol. 55:10108–10120. 34240846 10.1021/acs.est.1c03483

