# Supplementary material for: Multisectoral drivers of decarbonizing battery electric vehicles in China
Source: PNAS Nexus. 2023 May 16;2(5):pgad123. doi: 10.1093/pnasnexus/pgad123 (PMC10187665; doi:10.1093/pnasnexus/pgad123)
Supplement: pgad123_Supplementary_Data [file pgad123_supplementary_data.zip › PNASNEXUS-PNASNEXUS-2022-01146-T-s02.docx]

**Supporting Information for**

Multi-sectoral drivers of decarbonizing battery electric vehicles in China

Fang Wang^1^, Shaojun Zhang^1,2,3*^, Yinan Zhao^1^, Yunxiao Ma^1^, Yichen Zhang^1^, Anders Hove^4^, Ye Wu^1,2,3^

^1^ School of Environment, State Key Joint Laboratory of Environment Simulation and Pollution Control, Tsinghua University, Beijing 100084, P. R. China

^2^ State Environmental Protection Key Laboratory of Sources and Control of Air Pollution Complex, Beijing 100084, P. R. China

^3^ Beijing Laboratory of Environmental Frontier Technologies, School of Environment, Tsinghua University, Beijing, 100084, China

^4^ GIZ and Research Associate, Oxford Institute for Energy Studies

* Corresponding author: Shaojun Zhang.

**Email:**  zhsjun@tsinghua.edu.cn

Supporting Information Text

The drivers of decarbonizing LFP-BEVs from 2015 to 2030, which is the most important result of this paper as the counterpart of NCM-BEVs in the manuscript, is first shown in Fig. S1. Then a framework of the whole system boundary is pictured in Fig. S2, followed by detailed description of key parameters in well-to-wheels and vehicle cycle.

**Key well-to-wheels parameters**

***Real-world fuel consumption for ICEVs.*** The Dual Credit policy in China is promoting the improvement of ICEV efficiencies and the increasing penetration of BEV (1, 2). China’s fuel consumption regulations announce that the average fuel consumption for ICEVs achieved 7.0 L per 100 km and 6.5 L per 100 km by 2015 and 2020, respectively (3, 4). It is estimated the future level would decrease to 4.6 L per 100 km in 2030 and more grid-independent hybrid electric vehicles (HEVs) will penetrate to comply with this stringent target. However, many studies have revealed the significant discrepancies between official references and real-world fuel consumption for ICEVs in China. For example, a previous study noted a gap of 29% for the current MY (5), which was larger than the gap around 2015 (23% (6) by iCET). We consider the gap will not further increase, and thus average real-world fuel consumption levels are estimated to be 8.6 L per 100 km, 8.4 L per 100km, and 6.0 L per 100km, respectively. The values are not significantly different from the estimations in the previous studies (e.g., 8.0 L per 100km in Huo (7) and 8.04 L per 100km in Gan (8)). As for current HEV, we used 5.48 L per 100 km in 2020 based on investigations on existing vehicle models in China.

***Real-world fuel consumption for BEVs.*** The official reference electricity consumption for BEV is obtained from the annual report of China’s automobile industry (9). The fleet-average level dropped from 15.8 kWh per 100 km in 2016 to 12.5 kWh in 2020, representing a decrease of up to 20%. The official electricity consumption in 2030 is estimated to decrease to 11 kWh per 100 km. A few studies also reported real-world electricity consumption based on the operating monitoring data. For example, Zhao (10) derived real-world electricity consumption for massive BEVs in Beijing and Guangzhou, which were 17.1 and 16.7 kWh 100 km^-1^ in 2019, respectively. Otherwise as Table S1 shows, China’s fleet-average official electricity consumption of BEVs in 2019 (under the New European Driving Cycle, NEDC) is estimated to be 14.1 kWh 100km^-1^ based on the annual sales and the reported official electricity consumption for BEVs from 2015 to 2019. Therefore, we estimated the average road-to-lab gap in electricity consumption of BEVs to be 20%. The real-world electricity consumption of BEVs for the three scenario years is 19.0 kWh per 100 km (2015), 15.0 kWh per 100 km (2020), and 13.2 kWh per 100 km (2030), respectively.

***Well-to-tank and tank-to-wheels CO_2_ emissions of fossil fuels.*** Fossil fuels such as coal, natural gas, and petroleum fuels would be used in both the fuel cycle and vehicle cycle. Table S2 also summarized the well-to-tank (WTT; upstream extraction, transportation, and production processes) and tank-to-wheels (TTW; i.e., downstream combustion processes) CO_2_ emissions by fuel type based on GREET model with localized inputs. (11) For example, the WTT CO_2_ emissions for gasoline are 20.0, 19.1, and 17.2 g MJ^-1^ in 2015, 2020, and 2030, respectively, and the TTW CO_2_ emissions for gasoline are 69.2 g MJ^-1^. The lower carbon intensities of gasoline are attributed to the improved efficiency of the refinery industry and the cleaner electricity mix.

***Consumption-based regional electricity mixes and corresponding CO_2_ emission intensity.*** China’s power grid is composed of seven interconnected but relatively independent sub-grids: North, East, Central, Northeast, Northwest, Southwest, and South (see Fig. S3). Electricity mix refers to the proportion of generation type according to primary energy, including coal (Coal), natural gas (NG), hydropower (Hydro), nuclear (Nu), biomass (Bio), wind (Wind), and photovoltaics (Solar) in the power grid. This study estimated the generation-based electricity mix for each sub-grid based on the installed capacity and the annual utilization hours for each generation type, and then further estimated the consumption-based electricity mix by considering inter-region transmission (see Eqs. (1) and (2)).

$Mix_{y}=\frac{Cap_{y}\times Hour_{y}}{\mathrm{Gen}_{y}\times1000}\cdot T_{y}$ (1)

$Mix=\left[ \begin{matrix} Mix_{NG,N} & Mix_{NG,E} & \cdots& Mix_{NG,S} \\ Mix_{Coal,N} & Mix_{Coal,E} & \cdots& Mix_{Coal,S} \\ Mix_{Nu,N} & Mix_{Nu,E} & \cdots& Mix_{Nu,S} \\ Mix_{Bio,N} & Mix_{Bio,E} & \cdots& Mix_{Bio,S} \\ Mix_{Hydro,N} & Mix_{Hydro,E} & \cdots& Mix_{Hydro,S} \\ Mix_{Wind,N} & Mix_{Wind,E} & \cdots& Mix_{Wind,S} \\ Mix_{Solar,N} & Mix_{Solar,E} & \cdots& Mix_{Solar,S} \\ Mix_{Others,N} & Mix_{Others,E} & \cdots& Mix_{Others,S} \end{matrix} \right]$ (2)

$T=\left[ \begin{matrix} T_{N\to N} & T_{N\to E} & T_{N\to C} & T_{N\to NE} & T_{N\to NW} & T_{N\to SW} & T_{N\to S} \\ T_{E\to N} & T_{E\to E} & T_{E\to C} & T_{E\to NE} & T_{E\to NW} & T_{E\to SW} & T_{E\to S} \\ T_{C\to N} & T_{C\to E} & T_{C\to C} & T_{C\to NE} & T_{C\to NW} & T_{C\to SW} & T_{C\to S} \\ T_{NE\to N} & T_{NE\to E} & T_{NE\to C} & T_{NE\to NE} & T_{NE\to NW} & T_{NE\to SW} & T_{NE\to S} \\ T_{NW\to N} & T_{NW\to E} & T_{NW\to C} & T_{NW\to NE} & T_{NW\to NW} & T_{NW\to SW} & T_{NW\to S} \\ T_{SW\to N} & T_{SW\to E} & T_{SW\to C} & T_{SW\to NE} & T_{SW\to NW} & T_{SW\to SW} & T_{SW\to S} \\ T_{S\to N} & T_{S\to E} & T_{S\to C} & T_{S\to NE} & T_{S\to NW} & T_{S\to SW} & T_{S\to S} \end{matrix} \right]$ (3)

where *y* is the MY; *Mix* and *Cap* are expressed in matrix format as Eq. (3) and denote electricity mix (%) and capacity (GW) for each sub-grid, respectively; *Hour* (h) is a column vector for annual utilization hours of different generation types; *Gen* (TWh) is a row vector for total generation of each sub-grid; *T* is the electricity transmission matrix (%) among sub-grids as Eq (4). Data regarding electricity generation and transmission in 2015 and 2020 were obtained from China Electricity Statistical Yearbook (12). Regional capacity and annual utilization hours in future years were obtained from the Global Energy Interconnection Development and Cooperation Organization (GEIDCO)’s estimates (13). Data can be obtained from Dataset S1.

To estimate the CO_2_ emission intensities of different sub-grids with their electricity mixes, the intensity of each non-zero emission generation technology was processed. Thermal power generation technologies (i.e., coal-fired and NG-fired electricity) were selected first, whose CO_2_ emission intensity was calculated as *EF_thermal_*:

$EF_{thermal}=\frac{EF_{WTT}+\left( \frac{3.6}{Eff_{generation}}\times C_{ratio}\times44/12 \right)}{\left( 1-loss \right)}$ (4)

where *EF_WTT_* (g kWh^-1^) refers to the emission factor for process fuel production in the upstream processes (e.g., coal extraction and production for coal-fired electricity), which can be found in Table S3; *Eff_generation_* is the generation efficiency for each technology; *C_ratio_* (g MJ^-1^) represents the typical carbon content of each process fuel and *loss* is the electricity transmission loss in the grids. As for the numbers, 3.6 means unit conversion from kWh to MJ, while 44 and 12 represents the molar mass for CO_2_ and C, respectively. The average *C_ratio_* for coal in China was 25.9 g MJ^-1^ (25.2~27.0 g MJ^-1^) according to an investigation of 16 generating units of a totally 6.2 GW capacity. The value can be further confirmed by a previous study on Chinese coals (14). The *C_ratio_* of natural gas is estimated to be 15.2 g MJ^-1^. Table S3 gives the efficiency for overall thermal power and the transmission loss in 2015 and 2020 (with a typical value of 5.62% in 2020), both of which were obtained from the China Electricity Statistical Yearbook (12,15). The average energy consumption of thermal power plants with a capacity larger than 6 MW was 315 and 306 g of coal equivalent kWh^-1^ in 2015 and 2020 (namely 38.9% and 40.2% as efficiency), respectively. The efficiency of NG-fired electricity was obtained from the China Energy Statistical Yearbook (16), which is also consistent with an investigation of industrial standards conducted by the China Electricity Council (17). The efficiency of coal-fired electricity then can be separated from the overall thermal power. Parameters in 2030 are expected to be moderately improved. Apart from NG-fired and coal-fired electricity, the CO_2_ emission intensity for the other two non-zero emission technologies (i.e., biomass and nuclear electricity) were estimated based on the GREET model with localized inputs (11).

Based on the emission factors for various generation technologies, the life-cycle CO_2_ emission intensity of electricity in different sub-grids can be calculated according to their respective consumption-based power mixes, and results are shown in Table S4 and Fig. S3. More details can be found in Dataset S1.

**Key vehicle cycle parameters: curb weight**

***Basic information for vehicle curb weight.*** The average curb weight (CW) of passenger cars has increased recently from 1364 kg in 2015 to 1491 kg in 2020, according to MIIT (3,4). Among all the passenger cars, the CW data of BEVs were obtained according to the "Catalogue of Models of New-Energy Automobiles Exempt from Vehicle Acquisition Tax", which is 1292 kg in 2015 and 1535 kg in 2020, respectively. For the other part, the CW data of ICEVs were 1365 kg in 2015 and 1489 kg in 2020, calculated as Eqs. (5):

$CW_{\mathrm{ICEV}}=\frac{CW_{\mathrm{cars}}\times Sales_{\mathrm{vehicle}}-CW_{\mathrm{BEV}}\times Sales_{\mathrm{BEV}}}{\mathrm{Sale}s_{\mathrm{vehicle}}-Sales_{\mathrm{BEV}}}$ （5）

Where relative inputs can be found in Table S5. As for 2030, CW data for BEVs (1341 kg for NCM-BEVs and LFP-BEVs) and ICEVs (1297 kg) are referenced to the GREET model.

**Key vehicle cycle parameters: Major automotive metals**

***Life-cycle inventory data for major automotive metals (steel, aluminum, and copper).*** Some studies have conducted LCA analysis on crude steel, crude aluminum, and crude copper in China (18-22). However, crude metals should be further processed before meeting the specific needs of automotive metals, thus resulting in additional fossil fuel consumption and C2G emissions impact. For instance, body steel is obtained through processes including pickling, hot-rolling, cold-rolling, and galvanizing after crude metal production due to the higher requirements on processability and surface flatness.

We first updated the life-cycle inventory data of crude metals (23) by investigating the operation data from seven iron and steel plants, 70 electrolytic aluminum plants, and three copper smelters across the past years. Based on these data, the comprehensive energy consumption index given by their industry associations can be allocated to each process fuel in each subprocess. Next, we collected operation data of several case plants on both upstream (e.g., ore extraction, production of alumina, anode manufacture) and downstream sides (processing of crude metals) of the industrial chain, where recycled materials were considered as well. The life-cycle CO_2_ emission intensity per tonne of automotive metal in the three MY is calculated as:

$E_{metal,i,y}=P_{i,y}\times\sum_{j} (EF_{pri,j,i,y}\times Con_{pri,j,i,y})+(1-P_{i,y}）\times\sum_{j} (EF_{sec,j,i,y}\times Con_{sec,j,i,y})$ (5)

where *y* is the MY; *i* represents steel, aluminum, and copper; *j* represents the raw materials or process fuels during the production; *P* refers to the proportion of primary metal materials used while *(1-P)* for low-carbon metal materials, namely recycled materials in this study, which can be found in Table S6. *EF* and *Con* are emission factors and consumption of raw materials or process fuels during the production, respectively. The life-cycle CO_2_ emissions results can be found in Dataset S1.

***Life cycle inventory data of steel production in China.*** Steel is widely used in vehicle body structures, doors, powertrain systems, suspension systems and other vehicle components. Crude steel is mainly produced by two categories of technology in China: blast furnace-basic oxygen furnace (BF-BOF) and scrap-based electric arc furnace (EAF). As Fig. S4 shows, BF-BOF technology mainly takes iron ore as feedstock. Powdery iron ore is first agglomerated to form ether sinter or pellets, which can be further converted to crude iron in a blast furnace with the blending of coke and limestone (CaCO_3_). Next in the steelmaking process, crude iron together with a small fraction of scrap steel and alloy steel (12% in 2019 (24)) are added to adjust the ratios of trace elements (e.g., Mn, Cr, Mo, Si, etc.) for different purposes, which are transformed into crude steel in the basic oxygen furnace. EAF technology can ideally rely on scrap steel as the only feedstock; however, the actual proportion of scrap steel was only 51% in 2019 (24) and the rest of the feedstock input was purchased crude iron. In 2030, the penetration of scrap steel in EAF feedstock is assumed to be 100%.

For the BF-BOF technology, seven steel plants in China were investigated based on our first-hand surveys (four plants in Shandong Province) and literature review (25,26). Process fuels and raw materials consumption of each stage were accounted for first, and by-products including coke oven gas (COG) from the coke plant, blast furnace gas (BFG) from the blast furnace, basic oxygen furnace gas (BOFG) from the basic oxygen furnace, tar, crude benzene, and steam were recorded as well. Two factors make the CO_2_ emissions calculation of crude steel more complicated than other materials. One is that multiple by-products such as COG, BFG, and BOFG can be reused as process fuels or to generate captive electricity that may directly arouse the concern of associated double-counting of CO_2_ emissions. The other one is that the purchase of raw materials such as coke, crude iron, and scrap steel may lead to the leakage of life-cycle CO_2_ emissions in the calculation. In this study, we treated the by-products as different carbon-contained process fuels whose net CO_2_ emissions were determined by their consumption or production. Furthermore, we included the CO_2_ emissions of purchased materials by external suppliers into our system boundary. We found that although the CO_2_ emissions in each sub-process could be quite different across different plants, the total energy consumption and life-cycle CO_2_ emissions per tonne of crude steel production had much smaller differences.

For the other processes on the upstream and downstream sides of steel production, the consumption data of process fuels were derived from the *China Steel Yearbook 2020* (24). The upstream side mainly includes the ore production process (24% using underground mining and 76% using open-pit mining, respectively) and the following beneficiation process. On the downstream side, crude steel would experience one or more processes (e.g., pickling, hot rolling, cold rolling, and galvanizing) before becoming qualified automotive steel. Notably, various automotive steel products have different performances to meet the specific requirements of various vehicle components. We used the proportions for hot-rolled, cold-rolled, and galvanized steel of 21%, 19, and 60%, respectively, according to the GREET model. The CO_2_ emission factors of process fuels can be found in Table S2, while the CO_2_ emission factors of raw materials and by-products are presented in Dataset S1. The change in the CO_2_ emission intensity of automotive steel from 2020 to 2030 majorly reflects the increased penetration of scrap steel in EAF. The increased share of EAF, and the lower CO_2_ emission intensity of electricity generation.

***Life cycle inventory data of aluminum production in China.*** Automotive aluminum is a lightweight material competing with conventional steel and is also indispensable for Li-ion batteries. Crude aluminum is predominately produced with the electrolytic aluminum process. As Fig. S5 shows, bauxite is processed into alumina with the presence of NaOH, and then alumina is poured into special reduction cells with molten cryolite at 950℃, where the anode is made from pet coke is consumed and liquid primary aluminum is formed under strong electrical currents. Through a pipe, liquid primary aluminum is sucked into the vacuum bucket that will further be brought to the cast house for primary ingot casting. Aluminum alloys are made by mixing primary aluminum (or scrap aluminum) with various other metals (e.g., B, Fe, Si, Mg, Mn, Cu, Ni, Pb, Ti, Cr, Zn) for specific needs. Those alloys can be cast to the required form (Cast Aluminum) or rolled into aluminum strips and foil with a standardized shape (i.e., Wrought Aluminum).

The electrolysis process is the most energy-intensive process, which consumes 6.8% of the national electricity consumption. Regarding this process, the national-average comprehensive electricity consumption was officially reported to be 13,543 kWh t^-1^ in 2020 (27). Other process fuels such as natural gas and diesel were counted as well, and the average consumption of raw materials is estimated to be 1.90 t t^-1^ for aluminum and 0.40 t t^-1^ for the anode. For a better understanding of the technological levels of various aluminum electrolysis plants in China, we investigated a total of 70 plants and collected the direct-current electricity consumption (without considering a loss of around 350 kWh t^-1^) from the public announcements by the provincial department of industry and information technology. The samples are well represented. For example, among the 70 samples of plants, 26 plants are with the available data for 2020 (9 in Guangxi and 17 in Inner Mongolia), covering over eight million tons of primary aluminum production (approximately 22% of total production in China). Based on these samples, the average value was 13,196 kWh t^-1^, quite close to the official value (13,186 kWh t^-1^). Therefore, it is reasonable to estimate that in 2030 the average comprehensive electricity consumption and anode usage will reach the current advanced level, which are 13000 kWh t^-1^ and 0.38 t t^-1^, respectively. These estimations are also consistent with a related study (28).

The upstream and downstream processes of the aluminum industry are more complicated than the steel industry because the processes are undertaken by different well-specialized enterprises. We depicted different processes with available case data or other sources, such as the bauxite mining process from the GREET model, alumina refining process from Longkou Donghai Alumina Co., Ltd., cast aluminum production from an aluminum alloy casting hub plant in Zhejiang, and wrought aluminum production from Fujian Chalco Ruimin Co., Ltd., Henan Wanji Aluminum Foil Co., Ltd., Henan Zhongfu Industrial Co., Ltd., and Shandong Nanshan Aluminum Co., Ltd.

Emission factors for raw materials are presented in Dataset S1. It is worth noting that a high proportion of electricity consumed in the aluminum electrolysis process is provided by captive coal-fired power plants (74% in 2015 and 63% in 2020). Thus, the CO_2_ emission intensity of electricity consumption cannot directly be adopted as the national average or even sub-grid region values. We derived the specific electricity mix in the plants and estimated the corresponding CO_2_ emission intensities in Dataset S1.

***Life cycle inventory data of copper production in China.*** Automotive copper is used in various vehicle components such as gearbox, electric motors, vehicle electronics, and battery systems. Copper is produced either through pyrometallurgical or hydrometallurgical technologies. In this study, we focused on the pyrometallurgical route since hydrometallurgical technology could only account for a low amount of copper production. As Fig. S6 shows, the first process is copper beneficiation with copper ore as feedstock, where the copper content would increase from 0.5%~3% to 20%~35%. Copper concentrate is then processed in flash or bath smelting furnaces to obtain blister copper melt that has a high copper content (no less than 97.5%). To meet the need for higher purity, blister copper or scrap copper is further purified to be copper anode through processes including fire refining and electrolysis. Copper cathode (>99.9%) is further processed into the required shape for the automotive industry. That is, every time a process alongside the industrial chain is completed where energy and auxiliary materials are consumed, the copper content of the intermediate product would increase.

Focusing on the intermediate copper products produced after copper beneficiation, the total energy consumption for each product including blister copper, copper anode, and copper cathode was assumed according to the relevant industrial standards (GB 21248-2014). We also estimated that the future performance would be the same as the advanced level in 2020. To allocate the total energy consumption into each process fuel, we obtained data from three primary copper plants that produce blister copper with flash smelting (a plant in Inner Mongolia), copper anode (29), and copper cathode (ZiJin Copper Co., Ltd. (30)), respectively. Process fuel consumption, especially the proportions of the total energy consumption, was investigated including residual oil, diesel, LPG, NG, bituminous coal, anthracite, coke, electricity, and steam produced by coal boilers. Finally, the energy consumption proportion of Zijin Copper Co., Ltd. was selected for primary automotive copper while that of the plant in Inner Mongolia was selected for recycled automotive copper (see Dataset S1).

The upstream side of the copper production produces copper beneficiation from copper ore (42% using underground mining and 58% using open-pit mining, respectively (31)). We collected data from two plants that conducted copper beneficiation using the above two types of ore (Peyziwat County Tonghui Mining Industry Co., Ltd. and Anqing Copper Mining of Tongling Nonferrous Metals Group Co., Ltd.), and we have verified the reasonability with the industrial standard (YST 693-2009). The weighted CO_2_ emissions of the two plants represent the upstream side. Regarding the downstream side of the copper production chain, we obtained two copper processing plants (both subsidiaries of Jiangxi Copper Corp.) and used the average CO_2_ emissions to represent the downstream side.

The copper content of each intermediate product is the key parameter determining the life-cycle CO_2_ emissions of primary copper. We used the copper contents of 0.57%, 21.55%, and 99.95%, respectively, for ore, copper beneficiation, and copper cathode according to the operational data in the investigated plants. The copper loss rate was set to be 2.5% according to the industrial standard. The total copper beneficiation and ore consumption levels would be 4.76 t t^-1^ and 181 t t^-1^, which are approximately consistent with a previous study (29).

**Key vehicle cycle parameters: Li-ion battery**

***Automotive Li-ion battery systems.*** Nickel-cobalt-manganese oxide (NCM) and lithium iron phosphate (LFP) are two major types of automotive Li-ion batteries employed in BEVs. NCM systems involve different precursors such as NCM_111_, NCM_523_, NCM_622_, NCM_811_, and even specially nickel-cobalt-aluminum oxide (NCA). The CO_2_ emissions for each type of battery are calculated as:

$E_{battery,i,y}=\sum_{i} \left[ P_{i}\times\sum_{j} (EF_{j,i,y}\times(Con_{raw,j,i,y}+Con_{fuel,j,i,y}) \right]\times\frac{BC\times1000}{ED}\times(1-Be_{recycle})$ (6)

Where *y* is the MY; *i* represents different batteries; *j* represents the raw materials or process fuel consumption during the production; *P* is the market sales of each battery; *EF* (Emission factor) for materials and process fuel is derived from the GREET model. *Con* here can be further categorized into raw materials consumption obtained from the GREET model as well as processes fuel consumption in battery manufacture covering five battery plants (including 2 LFP plants and 3 NCM plants) in operation in China; *ED* and *BC* are energy density (Wh kg^-1^) and battery capability (kWh), respectively. *Be_recycle_* refers to the C2G CO_2_ mitigation benefits in the recycling process.

***Market share, energy density and battery capability.*** NCM is the main battery type and have various precursors, including NCM_111_, NCM_523_, NCM_622_, NCM_811_, and nickel-cobalt-aluminum oxide (NCA). These precursors have various mass fractions of active metals and show different energy density levels. In general, high-nickel-content precursors (in particular NCM_811_) are expected to represent the direction of battery improvements due to the advantage in energy density. The market share inter within batteries can be found in Table S7. For LFP batteries, hydrothermal synthesis and solid-phase synthesis are the two main production pathways led by two leading suppliers (BTR and Dynanonic). Due to the lack of detailed data, we used the average of two pathways in the further calculation of LFP batteries. Notably, LFP batteries in passenger BEVs accounted for only 15% in 2020, but it has increased to approximately 40% in 2021.

We can obtain *ED* and *BC* on a national scale based on the vehicle information of the BEV market.

Regarding *ED*, the cell-level and pack-level *ED* were obtained from the annual report of China’s automobile industry (9). As Table S8 illustrates, the average pack-level energy density of NCM and LFP batteries were quite comparable, approximately 104 Wh kg^-1^ in 2016 and 145 Wh kg^-1^ in 2020, although the material-level and cell-level energy density levels of NCM are significantly higher than those of LFP. This is because the thermal management system of the NCM battery increases the non-effective weight at the pack level. Nevertheless, we acknowledge that there are a few uncertainties that can be further evaluated in the future. First, due to the variety of NCM precursors (NCM_111_, NCM_523_, NCM_622_, NCM_811_, NCA), the average ED might change significantly if one precursor becomes predominant in the future. Second, LFP batteries have been largely used in commercial BEVs, and the average ED of LFPs might differ from that of LFPs used in light-duty passenger electric vehicles. To address the impacts of the first concern, we further compared the average life-cycle CO_2_ emissions of NCM batteries by using unified and divergent ED for each type of precursor. As Table S9 illustrates, the differences in weighted average life-cycle CO_2_ emissions of NCM batteries are below 2%. Therefore, the simplification of the calculation for NCM batteries in this study is reasonable. For the second concern, the ED of LFP batteries loaded in commercial BEVs would achieve 160 Wh kg^-1^ while the value for passenger BEVs was no higher than 143 Wh kg^-1^ in 2020 according to "Catalogue of Models of New-Energy Automobiles Exempt from Vehicle Acquisition Tax". Thus, using the overall average ED of the LFP batteries would be not appropriate. We used the average ED of passenger LFP-BEVs in the catalogue, which is 130 Wh kg^-1^ in 2020, and the corresponding ED of NCM is calculated to be 148 Wh kg^-1^ based on the market share and the average value of 145 Wh kg^-1^. As a result, a unified ED of 103 Wh kg^-1^ was used for both LFP and NCM batteries in 2015. The future (2030) parameters of energy density are estimated to be 259 Wh kg^-1^ (NCM_811_) and 202 Wh kg^-1^ (LFP) based on a previous study (32). In addition, we used 180 Wh kg^-1^ (an NCM_811_ prismatic battery) and 140 Wh kg^-1^ (an LFP blade battery) to represent the present advanced ED levels for NCM and LFP batteries, and these two batteries have been loaded in BEV products in the market (XPeng G3 and BYD Yuan).

Regrading BC, the average *BC* of Li-ion batteries for MY 2015 was 33 kWh, and the weights of battery packs were approximately 320 kg. For MY 2020, the battery capability was 46 kWh, and the weights of NCM and LFP battery packs were 313 and 356 kg, respectively. This study estimates the fleet-average reference all-electricity range (AER) would increase to 500 km (417 km under real-world conditions), and thus a 55 kWh battery could represent the average requirement. The average battery weight for MY 2030 BEVs would be 212 kg for NCM and 272 kg for LFP.

***Consumption of raw materials and process fuels for Li-ion batteries.*** The raw materials consumption for different Li-ion batteries per kWh are given in the form of weight proportion (see Fig. S7), which were obtained from the GREET model (33). In the model, the weight proportion of each raw material such as active material for the cathode, carbon/graphite for the anode, aluminum/copper for the current collector, plastic for the separator, and electronic parts for the battery management system (BMS) is calculated based on a bottom-up design for a prismatic battery.

The consumption data of process fuels per kWh of Li-ion battery were investigated from five typical battery manufacture plants in China (2 LFP plants and 3 NCM plants) (see Fig. S8). Generally, natural gas (NG), electricity, circulating water, and steam are the four main process fuels in battery manufacture from raw materials to battery packs, where circulating water is driven by a motor pump while steam is generated with an NG boiler. These process fuels are needed in the battery manufacture steps including electrode preparation (active slurry mixing, coating on current collector, calendaring, slitting, drying), cell production (cutting, sintering, stacking, ultrasonic welding, enclosing, electrolyte filling), module assembly, and pack assembly (34). We were not able to get access to the detailed operation data for each process. In our data investigation, we collected the plant-level consumption data of various process fuels throughout the year. By further collecting the total battery production, we calculated fuel consumption per one kWh of Li-ion battery. For the two LFP plants, the energy consumption levels are close to each other. For the three NCM plants, the total energy consumption levels differ significantly, which is caused by the different levels of capacity utilization and technological advancement.

***Emission factors for raw materials and process fuels during Li-ion battery manufacture.*** Emission factors for raw materials are obtained from the GREET model (see Table S10) and emission factors of consuming process fuels during the production can be found in Table S2.

***Battery recycling.*** In the battery recycling system, reuse that extends the product life and recycling that avoids new primary material production are two main measures that affect C2G emissions of Li-ion batteries. Prior LCA studies have used divergent boundaries, functional units, environmental impact indicators, and recycling technologies (35-39). In this study, we considered recycling Li-ion batteries via hydrometallurgical recycling would predominate by 2030, and referred to the data reported in Jiang et al. (39) to estimate the C2G CO_2_ mitigation benefits for LFP (7.0%) and NCM (13.9%) batteries.

***Sensitivity analysis.*** Whereas the road-to-lab gap mentioned above may greatly affect the CO_2_ reduction benefits of BEVs, we conducted sensitivity analysis to address this issue (See Fig. S9). We selected 2020 as MY, and listed three grids with different carbon intensity. Providing a fixed 30% road-to-lab gap for ICEV, the C2G CO_2_ reduction benefits may decrease with enlarged road-to-lab gap of BEVs. Focusing on national grid, 41% reduction benefits occur at 20% gap while 38% reduction at 30% gap and 26% reduction at 70% gap. Further considering other grids, 30% and 53% reduction compared with ICEVs would be possible in north and south grid, respectively, at 20% gap. Even under extremely unfriendly circumstances with a road-to-lab gap of 70% for BEVs, driving BEVs in north grid could still achieve an 11% reduction.


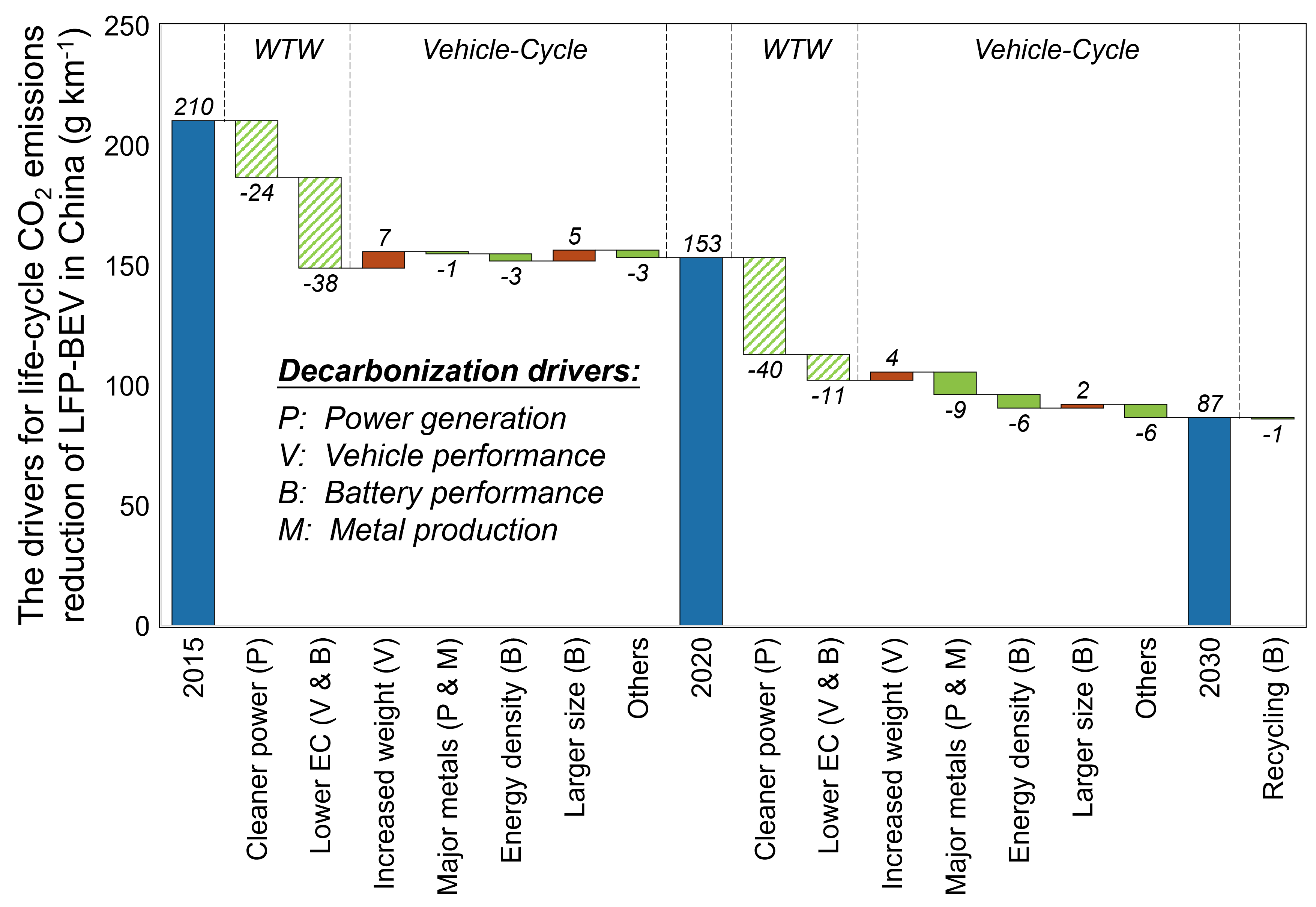


Fig. S1. Drivers of decarbonizing LFP-BEVs from 2015 to 2030. The drivers are categorized into two phases (WTW and vehicle-cycle) and four major parts: P: Power generation; V: Vehicle performance (e.g., vehicle weight, energy consumption (EC)); B: Battery performance (e.g., battery size, energy density); and M: Metal production.


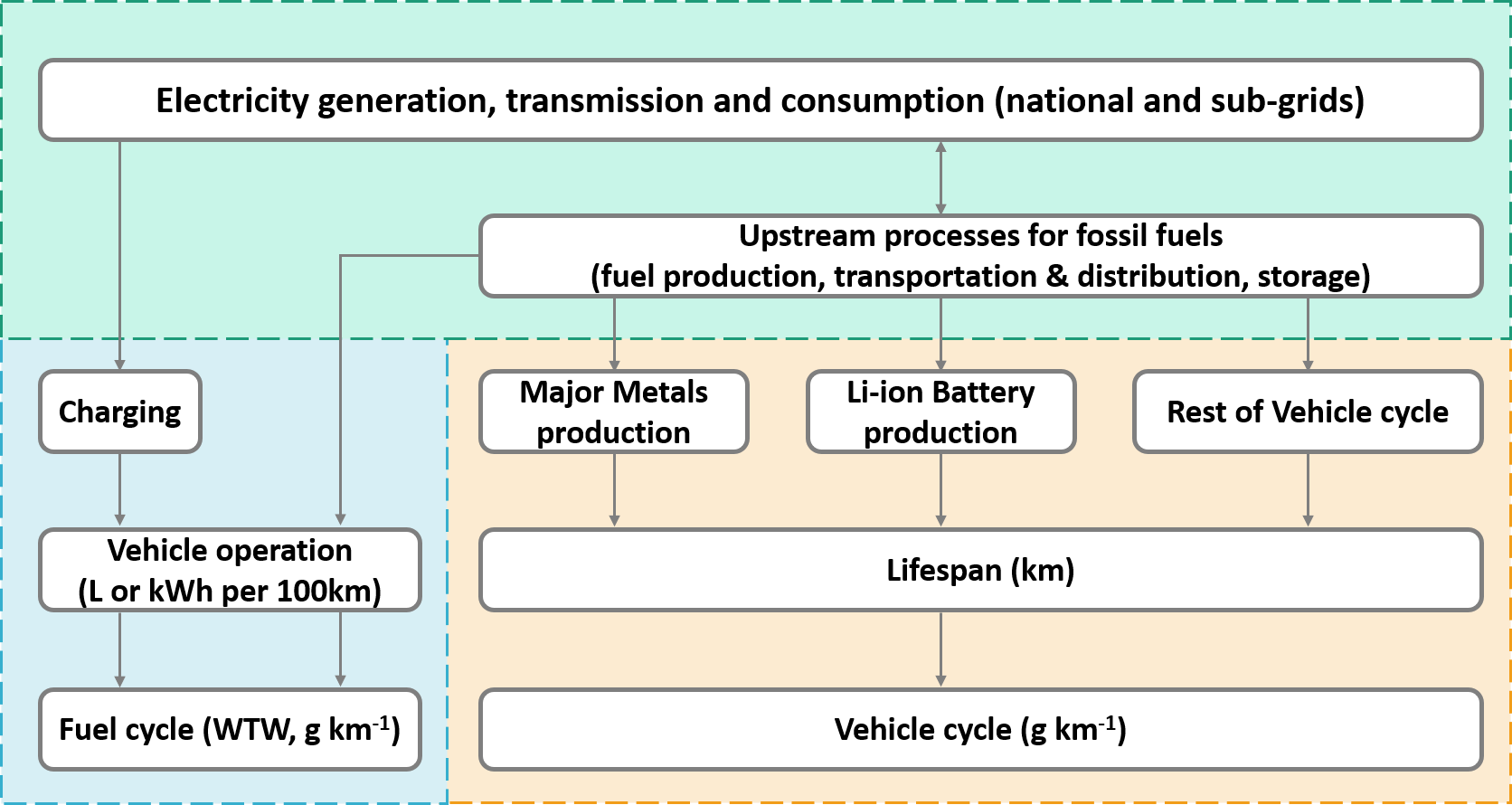


Fig. S2. Scope of cradle-to-grave analysis of CO_2_ emissions for BEVs in China


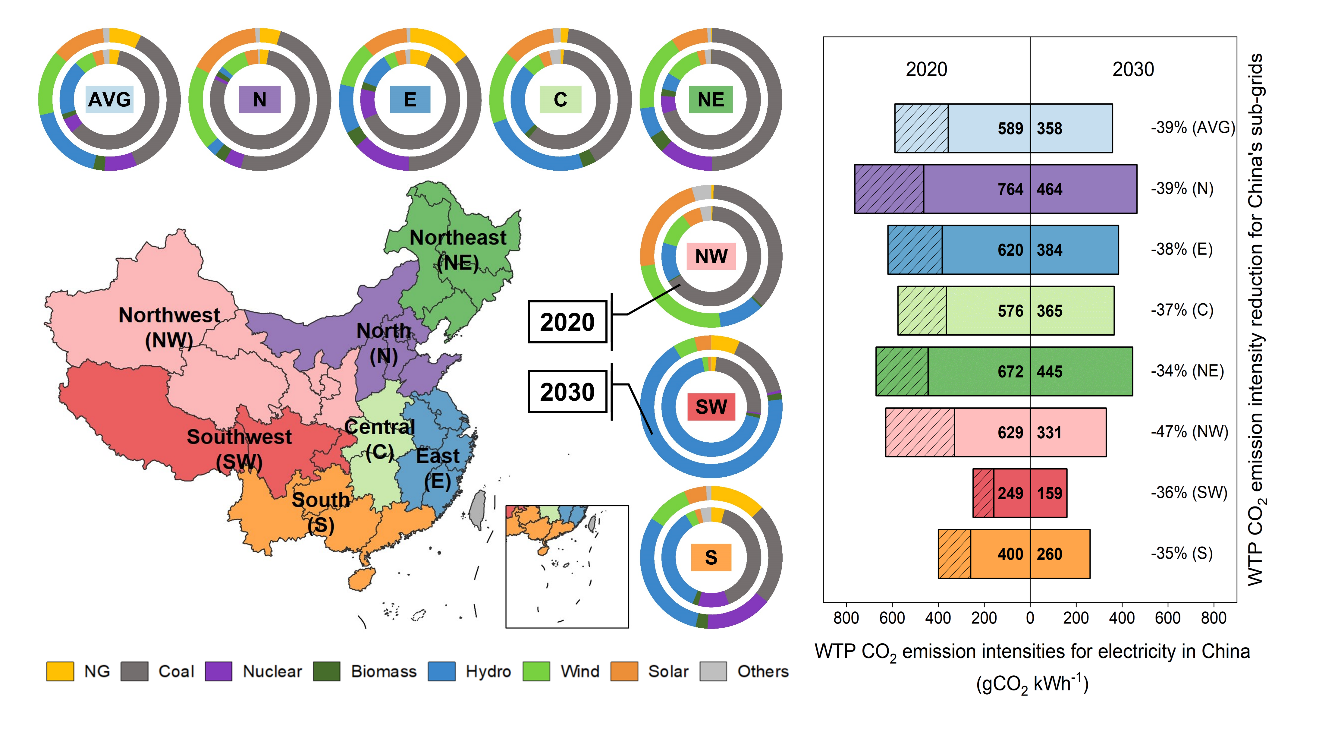


Fig. S3. Consumption-based electricity mixes and Well-to-Pump (WTP) CO_2_ emission intensities for the national average and all sub-grids in 2020 and 2030.


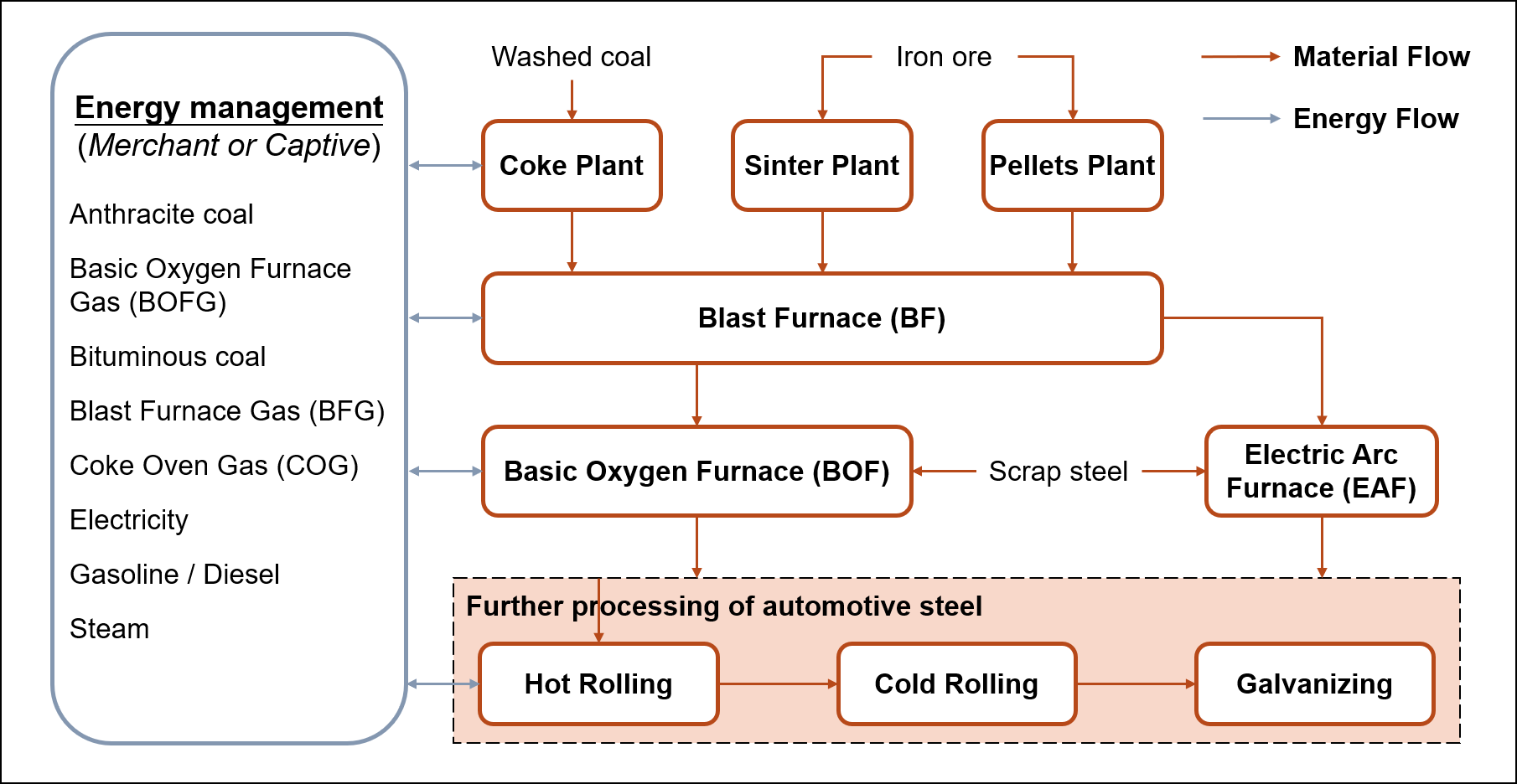


Fig. S4. The flowchart of industrial processes and feedstocks of steel-making in China.


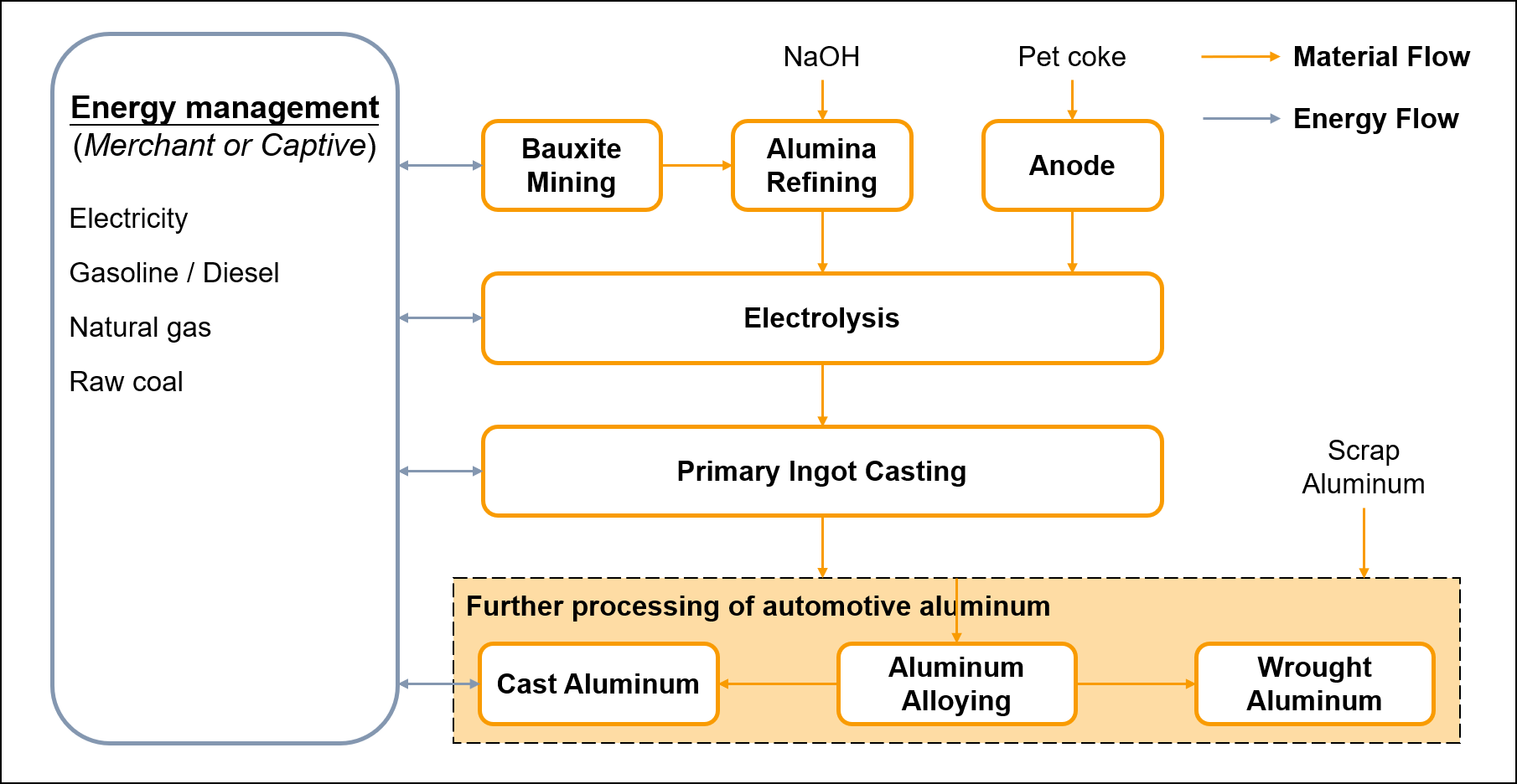


Fig. S5. The flowchart of industrial processes and feedstocks of cast aluminum and wrought aluminum production in China.


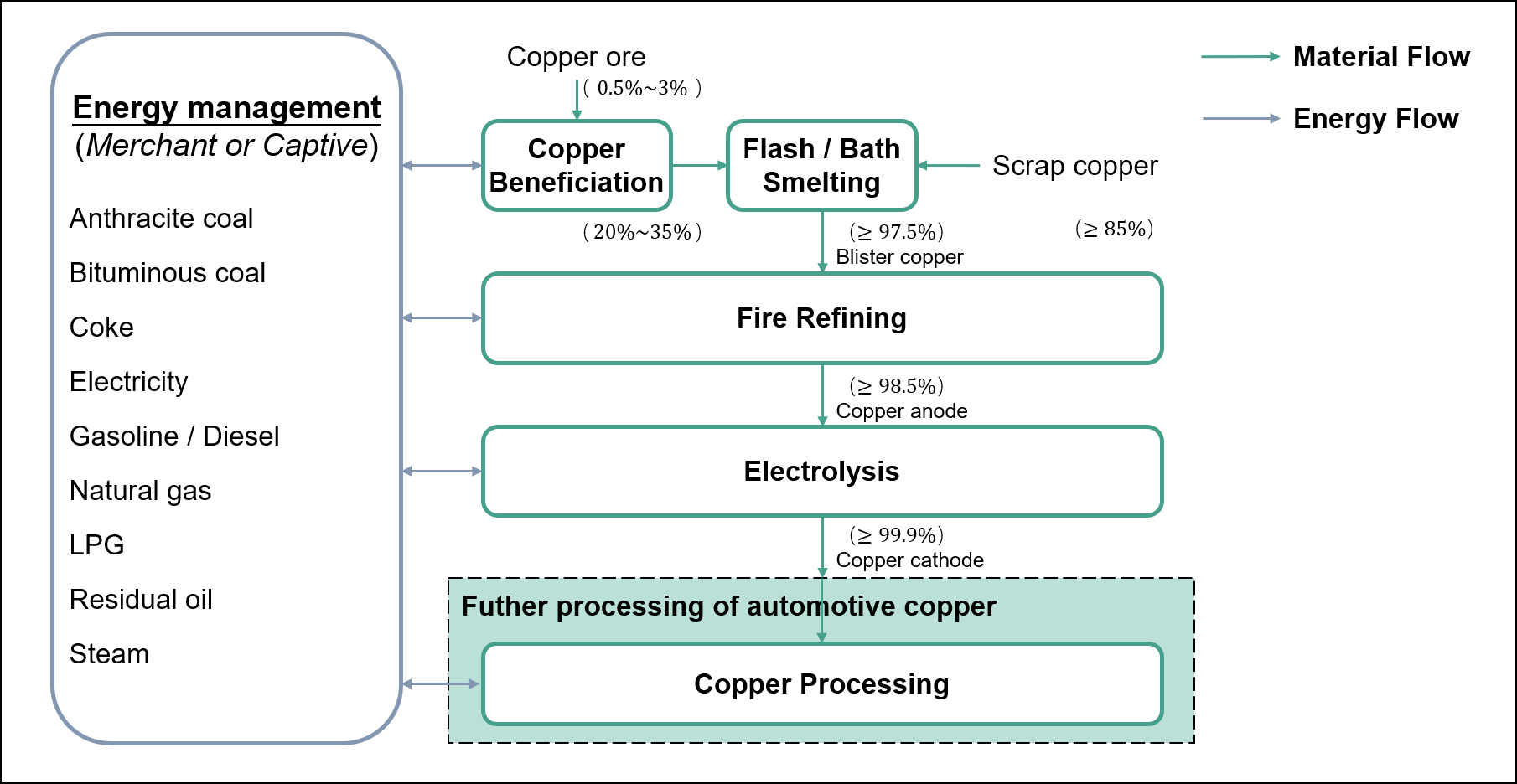


Fig. S6. The flowchart of industrial processes and feedstocks of copper production in China.


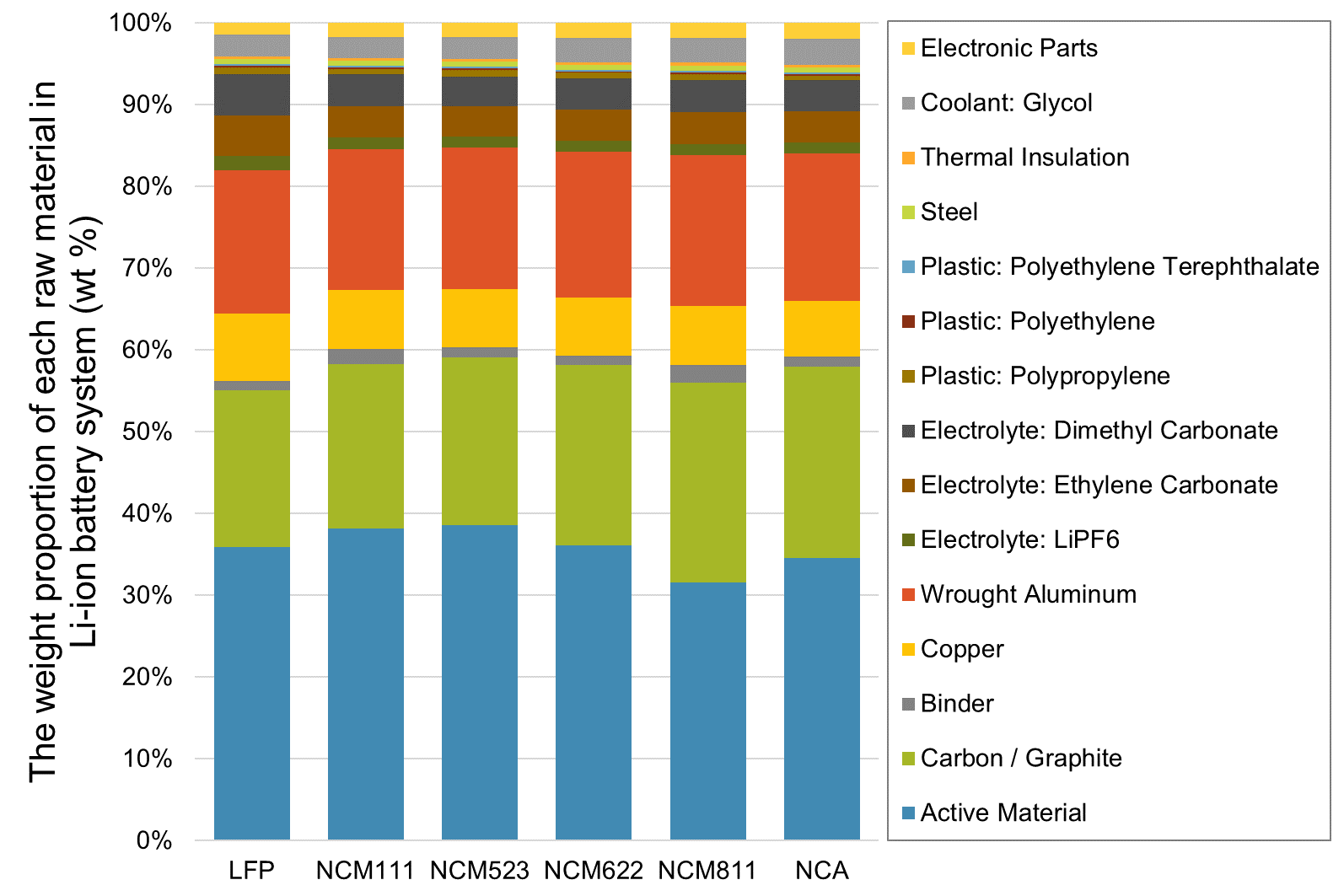


Fig. S7. The weight proportions of each raw material in various Li-ion battery systems (wt %), which were developed by referring to the GREET model.


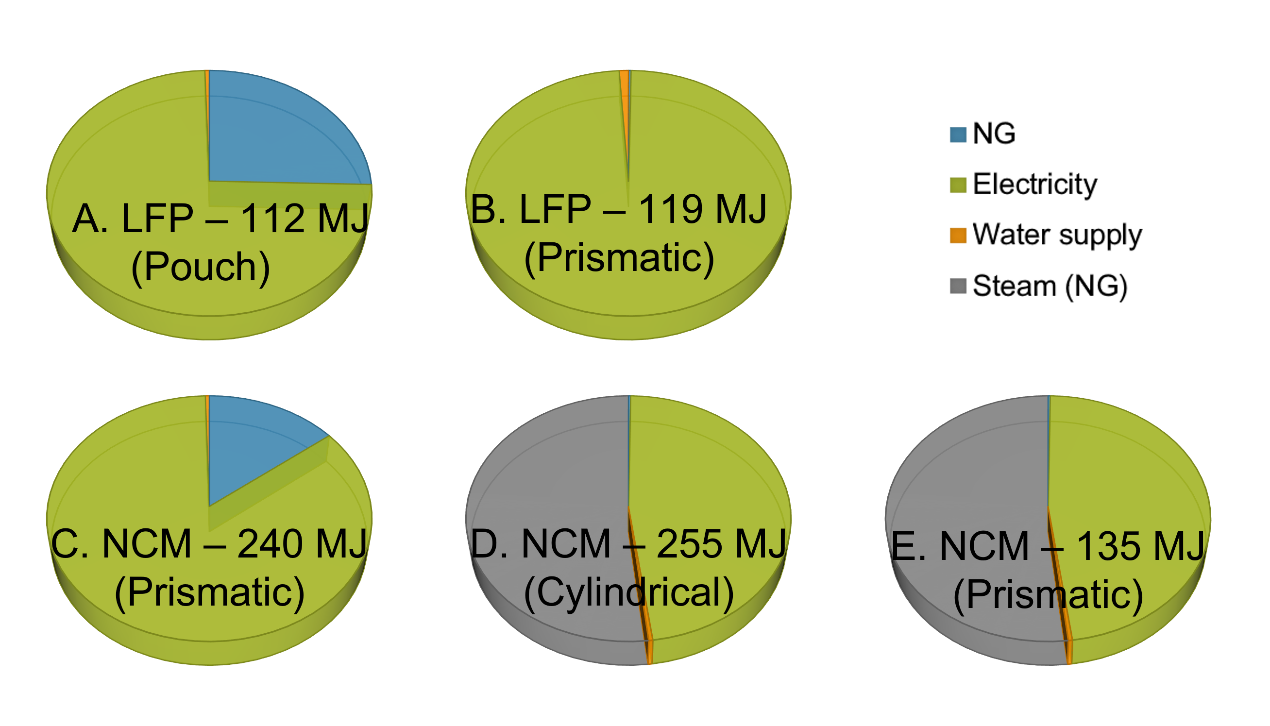


Fig. S8. Plant-level energy consumption of process fuels per kWh of battery manufacture (from raw materials to battery packs) for five battery plants in China.


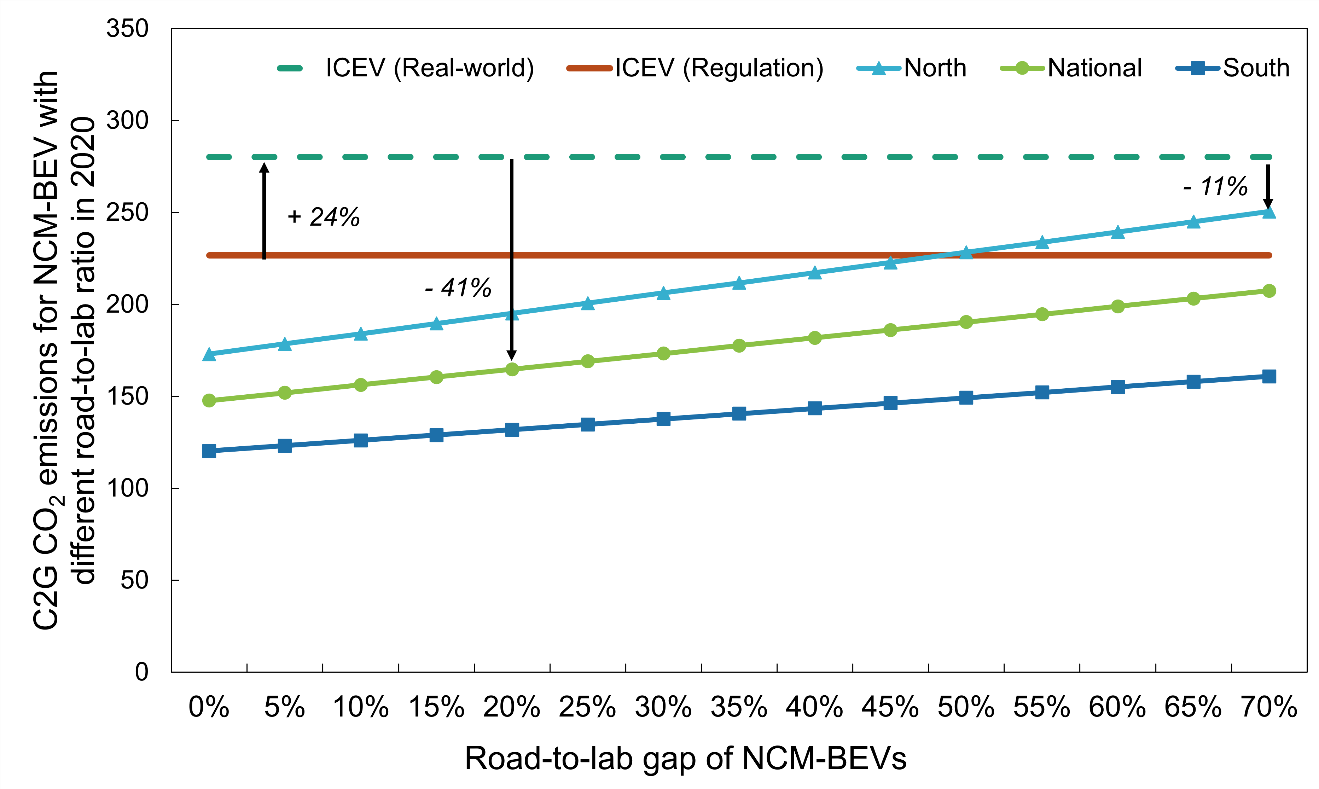


Fig. S9. C2G CO_2_ emissions for NCM-BEV with different road-to-lab ratios in 2020.

Table S1. Annual sales, official electricity consumption and estimated real-world electricity consumption of BEVs in China (2015-2020).

| Model year | Annual sales  (Million) | Official electricity consumption  (kWh 100km^-1^) | Estimated real-world electricity consumption  (kWh 100km^-1^) |
| --- | --- | --- | --- |
| 2015 | 0.25 | 15.86 | 19.0 |
| 2016 | 0.41 | 15.73 | 18.9 |
| 2017 | 0.65 | 14.28 | 17.1 |
| 2018 | 0.98 | 13.82 | 16.6 |
| 2019 | 0.97 | 13.28 | 15.9 |
| 2020 | 1.12 | 12.49 | 15.0 |
| 2019 fleet (calendar year) | - | - | ~17.0 (10) |

Note: The sales and official FC are reported in the annual report of China's automobile industry (9).

Table S2. Life-cycle CO_2_ emission intensity of major fossil fuels in China (g MJ^-1^) in 2015, 2020 and 2030.

|  | WTT  (2015) | WTT  (2020) | WTT  (2030) | TTW |
| --- | --- | --- | --- | --- |
| Coal | 4.0 | 3.8 | 3.3 | 94.6 |
| Coke | 4.0 | 3.8 | 3.3 | 102.0 |
| Bituminous coal | 4.0 | 3.8 | 3.3 | 94.6 |
| Anthracite | 4.0 | 3.8 | 3.3 | 99.0 |
| NG | 5.6 | 5.5 | 5.4 | 55.6 |
| Residual oil | 11.7 | 11.0 | 9.5 | 76.0 |
| Gasoline | 20.0 | 19.1 | 17.2 | 69.2 |
| Diesel | 15.6 | 14.8 | 13.1 | 74.3 |
| LPG | 17.4 | 16.6 | 14.8 | 59.8 |
| Pet Coke | 12.3 | 11.6 | 10.0 | 93.9 |
| Steam (Coal) |  |  |  | 113.5 |
| Steam (NG) |  |  |  | 78.5 |

Note: Data are derived from the GREET model with localized inputs (15).

Table S3. Generation efficiency and electricity transmission loss for calculating life-cycle CO_2_ emission intensity of natural gas (NG)-fired and coal-fired electricity.

|  | 2015 | 2020 | 2030 |
| --- | --- | --- | --- |
| *Eff_thermal power_* | 38.9% | 40.2% | - |
| *Eff_NG-fired_* | 54.7% | 53.0% | 55.0% |
| *Eff_coal-fired_* | 38.3% | 39.5% | 41.5% |
| *loss* | 6.64% | 5.62% | 4.80% |

Note: Data are obtained from the China Electricity Statistical Yearbook (12, 15) and China Energy Statistical Yearbook (16).

Table S4. Life-cycle CO_2_ emission intensity of electricity generation by energy type and sub-grid region (g kWh^-1^), including WTT CO_2_ emissions such as coal extraction and transportation for coal-fired electricity generation.

|  |  | 2015 | 2020 | 2030 |
| --- | --- | --- | --- | --- |
| By energy type | Coal-fired | 996 | 952 | 892 |
|  | NG-fired | 437 | 445 | 424 |
|  | Biomass | 36 | 33 | 32 |
|  | Nuclear | 11 | 8 | 4 |
| National average | | 689 | 589 | 358 |
| By sub-grid region | North | 914 | 773 | 472 |
|  | East | 802 | 640 | 400 |
|  | Central | 689 | 571 | 369 |
|  | Northeast | 843 | 671 | 445 |
|  | Northwest | 753 | 629 | 331 |
|  | Southwest | 218 | 238 | 153 |
|  | South | 488 | 398 | 260 |

Table S5. Curb weight for BEVs and ICEVs from 2015 to 2020.

|  | Total vehicle sales ^a^  (Million) | CW, passenger cars ^b^ (kg) | CW, ICEV (kg) | CW, BEV ^c^ (kg) |
| --- | --- | --- | --- | --- |
| 2015 | 24.60 | 1364 | 1365 | 1292 |
| 2016 | 28.03 | 1392 | 1394 | 1234 |
| 2017 | 28.88 | 1419 | 1420 | 1363 |
| 2018 | 28.08 | 1438 | 1439 | 1407 |
| 2019 | 25.77 | 1461 | 1459 | 1507 |
| 2020 | 25.31 | 1491 | 1489 | 1535 |

Notes:

1. Data from the annual report of China's automobile industry. (9)
2. Data from the Announcement on the Corporate Average Fuel Consumption (CAFC) and New Energy Vehicle (NEV) Credits of Chinese Passenger Car Manufacturers from 2015 to 2020. (3,4)
3. Data from the Catalogue of Models of New-Energy Automobiles Exempt from Vehicle Acquisition Tax.

Table S6. The proportion of metal production technologies in China.

|  | 2015 | 2020 | 2030 |
| --- | --- | --- | --- |
| Steel (BF-BOF^a^) | 95.3% | 94.9% | 79.2% |
| Steel (EAF^b^) | 4.7% | 5.1% | 20.8% |
| Aluminum (Primary) | 85.0% | 83.0% | 74.0% |
| Aluminum (Recycled) | 15.0% | 17.0% | 26.0% |
| Copper (Primary) | 63.6% | 67.6% | 83.1% |
| Copper (Recycled) | 36.4% | 32.4% | 16.9% |

Notes:

1. BF-BOF refers to blast furnace – basic oxygen furnace technology;
2. EAF refers to scrap-based electric arc furnace technology.

Table S7. Market sales for different NCM precursors.

|  | 2015 | 2018 | 2019 | 2020 |
| --- | --- | --- | --- | --- |
| NCM_111_ | 26% | 7.1% | 4.8% | 0.7% |
| NCM_523_ | 72% | 55.1% | 49.3% | 42.4% |
| NCM_622_ | 1% | 20.4% | 22.9% | 31.4% |
| NCM_811_ | 0.1% | 8.5% | 11.4% | 17.7% |
| NCA | 1% | 8.9% | 11.7% | 7.8% |

Note: Market sales data in 2018-2020 are obtained from a commercial website ([www.smm.cn](http://www.smm.cn)), and the data in 2015 were estimated based on the understanding of the battery industry. In 2030, we assume NCM_811_ would become the only NCM precursor to achieve the high requirement for energy density.

Table S8. Battery capability and energy density for Li-ion battery from 2015 to 2020.

| Model year | BC^a^  (market data)  (kWh) | Battery ED^a^  (market data)  (Wh kg^-1^) | Cell-level ED^b^ (NCM) (Wh kg^-1^) | Pack-level ED^b^ (NCM) (Wh kg^-1^) | Cell-level ED^b^ (LFP)  (Wh kg^-1^) | Pack-level ED^b^ (LFP) (Wh kg^-1^) |
| --- | --- | --- | --- | --- | --- | --- |
| 2015 | 33 | 103 | - | - | - | - |
| 2016 | 32 | 104 | 166 | 104 | 132 | 107 |
| 2017 | 27 | 109 | 167 | 106 | 143 | 114 |
| 2018 | 39 | 133 | 192 | 127 | 160 | 133 |
| 2019 | 47 | 148 | 203 | 136 | 166 | 140 |
| 2020 | 46 | 145 | 213 | 145 | 169 | 143 |

Note:

1. Data are obtained by calculation based on annual sales and their corresponding parameters.
2. Data are obtained from "Annual report of China's automobile industry." (9)
3. The pack-level energy densities of LFP batteries are higher than NCM batteries, which could be explained by blending of LFP batteries applied in commercial BEVs in statistics. Those batteries could achieve higher energy density. The final ED parameters for NCM and LFP in this study are 103 and 103 Wh kg^-1^ (2015), and 148 and 130 Wh kg^-1^ (2020).

Table S9. Comparison of life-cycle CO_2_ emissions of different NCM batteries using the two sets of battery energy density values.

| Model year | Type | Share^a^ | Average ED  (Wh kg^-1^) | life-cycle CO_2_ emissions^b^  (kg kWh^-1^) | Divergent ED  (Wh kg^-1^) | life-cycle CO2 emissions^c^  (kg kWh^-1^) |
| --- | --- | --- | --- | --- | --- | --- |
| 2015 | NCM_111_ | 25.7% | 103 | 155 | 85 | 188 |
|  | NCM_523_ | 71.8% | 103 | 159 | 110 | 149 |
|  | NCM_622_ | 1.4% | 103 | 159 | 140 | 117 |
|  | NCM_811_ | 0.1% | 103 | 159 | 155 | 106 |
|  | NCA | 1.0% | 103 | 159 | 150 | 109 |
|  | **Weighted Average** | | **103** | **158** | **104** | **158** |
| 2020 | NCM_111_ | 0.7% | 145 | 109 | 100 | 159 |
|  | NCM_523_ | 42.4% | 145 | 112 | 125 | 130 |
|  | NCM_622_ | 31.4% | 145 | 112 | 155 | 105 |
|  | NCM_811_ | 17.7% | 145 | 113 | 170 | 96 |
|  | NCA | 7.8% | 145 | 113 | 165 | 99 |
|  | **Weighted Average** | | **145** | **112** | **145** | **114** |

Note:

1. Sales information is listed in Table S7.
2. Life-cycle CO_2_ emissions of NCM battery with average ED inputs.
3. Life-cycle CO_2_ emissions of NCM battery with divergent ED inputs.
4. The mission of this table is to test how much the final weighted average life-cycle CO_2_ emissions would change when we select average ED or divergent ED. And the results show little differences in 2015 and 2020.

Table S10. CO_2_ emission factors for raw materials in Li-ion batteries (Unit: t CO_2_ t^-1^)

|  | 2015 | 2020 | 2030 |
| --- | --- | --- | --- |
| Active material (NCM_111_) | 20.2 | 19.0 | 16.1 |
| Active material (NCM_523_) | 21.0 | 19.9 | 17.0 |
| Active material (NCM_622_) | 21.9 | 20.9 | 18.0 |
| Active material (NCM_811_) | 24.2 | 23.2 | 20.0 |
| Active material (NCA) | 25.7 | 24.6 | 21.3 |
| Active material (LFP_Hydrothermal_) | 4.9 | 4.8 | 4.7 |
| Active material (LFP_Solid-phase_) | 3.0 | 2.8 | 2.4 |
| Carbon / Graphite | 4.6 | 4.4 | 4.2 |
| Binder | 2.8 | 2.5 | 1.9 |
| Copper | 5.7 | 5.1 | 3.7 |
| Wrought Aluminum | 15.6 | 14.1 | 10.0 |
| Electrolyte: LiPF6 | 16.5 | 14.1 | 8.7 |
| Electrolyte: Ethylene Carbonate | 0.4 | 0.4 | 0.3 |
| Electrolyte: Dimethyl Carbonate | 1.5 | 1.4 | 1.2 |
| Plastic: Polypropylene | 2.0 | 1.9 | 1.6 |
| Plastic: Polyethylene | 2.5 | 2.3 | 1.9 |
| Plastic: Polyethylene Terephthalate | 3.2 | 3.0 | 2.6 |
| Steel | 2.2 | 2.2 | 1.8 |
| Thermal Insulation | 2.1 | 2.0 | 1.6 |
| Coolant: Glycol | 1.6 | 1.6 | 1.6 |
| Electronic Parts | 33.0 | 29.1 | 20.1 |

Dataset S1 (separate file)

Dataset S1 presents the detailed data of electricity mix, life cycle inventories (LCIs) of steel, aluminum, and copper production in China.

**SI References**

1. X. He, et al., Greenhouse gas consequences of the China dual credit policy. Nat. Commun. 11 (2020).
2. S. Ou, et al., China's vehicle electrification impacts on sales, fuel use, and battery material demand through 2050: optimizing consumer and industry decisions. iScience 24, 103375 (2021).
3. Ministry of Industry and Information Technology of China (MIIT), Data from "Announcement on the corporate average fuel consumption (CAFC) and new energy vehicle (NEV) credits of Chinese passenger car manufacturers in 2015.". (in Chinese)
4. Ministry of Industry and Information Technology of China (MIIT), Data from "Announcement on the corporate average fuel consumption (CAFC) and new energy vehicle (NEV) credits of Chinese passenger car manufacturers in 2020.". (in Chinese)
5. China Automotive Technology and Research Center (CATARC), Data from "CCRT research on fuel consumption and consumer satisfaction in China (2020).". (in Chinese)
6. Innovation Center for Energy and Transportation (iCET), Data from "2018 new data analysis on real-world driving and fuel consumption for passenger cars in China.".
7. H. Huo, Q. Zhang, M. Q. Wang, D. G. Streets, K. He, Environmental implication of electric vehicles in China. Environ. Sci. Technol. 44, 4856-4861 (2010).
8. Y. Gan, et al., Provincial greenhouse gas emissions of gasoline and plug-in electric vehicles in China: comparison from the consumption-based electricity perspective. Environ. Sci. Technol. 55, 6944-6956 (2021).
9. Ministry of Industry and Information Technology of China (MIIT), Data from "Annual report of China's automobile industry.".
10. Y. Zhao, Z. Wang, Z. M. Shen, F. Sun, Assessment of battery utilization and energy consumption in the large-scale development of urban electric vehicles. Proceedings of the National Academy of Sciences 118, e2017318118 (2021).
11. X. He, et al., Economic and climate benefits of electric vehicles in China, the United States, and Germany. Environ. Sci. Technol. 53, 11013-11022 (2019).
12. National Bureau of Statistics of China (NBSC), Data from "China Electric Power Yearbook 2021.".
13. Global Energy Interconnection Development and Cooperation Organization (GEIDCO), Data from “The ‘14th Five-Year Plan’ for China's Electricity Development.”.
14. E. Gao, et al., The average carbon content per unit energy in Chinese coals. Earth and Environment. 42, 95-101 (2014). (in Chinese)
15. National Bureau of Statistics of China (NBSC), Data from "China Electric Power Yearbook 2020.".
16. National Bureau of Statistics of China (NBSC), Data from "China Energy Statistical Yearbook 2020.".
17. China Electrical Council (CEC), Data from “Analysis of energy consumption index and consumption difference of gas generator sets 2018”. Available at

https://cec.org.cn/detail/index.html?3-179826 (in Chinese)

1. J. Chen, et al., Environmental benefits of secondary copper from primary copper based on life cycle assessment in China. Resources, Conservation and Recycling 146, 35-44 (2019).
2. J. Hong, J. Zhou, J. Hong, X. Xu, Environmental and economic life cycle assessment of aluminum-silicon alloys production: a case study in China. J. Clean. Prod. 24, 11-19 (2012).
3. X. Ma, et al., Life cycle assessment and water footprint evaluation of crude steel production: a case study in China. J. Environ. Manage. 224, 10-18 (2018).
4. T. Peng, X. Ou, X. Yan, G. Wang, Life-cycle analysis of energy consumption and GHG emissions of aluminium production in China. Energy Procedia. 158, 3937-3943 (2019).
5. W. Zhang, et al., Analyzing the environmental impact of copper-based mixed waste recycling-a LCA case study in China. J. Clean. Prod. 284, 125256 (2021).
6. X. He, et al., Cradle-to-gate greenhouse gas (GHG) burdens for aluminum and steel production and cradle-to-grave GHG benefits of vehicle lightweighting in China. Resources, Conservation and Recycling 152, 104497 (2020).
7. China Iron and Steel Association (CISA), Data from “China Steel Yearbook 2020.”. (in Chinese)
8. H. Liu, J. Fu, S. Liu, X. Xie, X. Yang, Calculation methods and application of carbon dioxide emission during steel-making process. Iron & Steel. 51, 74-82 (2016).
9. Z. Lu, H. Chen, Z. Hao, D. Hao, W. Li, Discussion on correction method of carbon dioxide emission in iron and steel enterprises. Henan Science 37, 1317-1323 (2019).
10. China Nonferrous Metals Industry Association (CNMIA), Data from “China's non-ferrous metal industry reports 2020.”. (in Chinese)
11. Y. Zhou, et al. Analysis and calculation of greenhouse gas emission reduction potential in aluminium electrolysis production process. Light Metals, 17-21 (2021).
12. W. Wang, et al. Carbon emission accounting method and strategy analysis under the background of double carbon: taking copper and aluminium industry as an example. Nonferrous Metals (Extractive Metallurgy). 1-12 (2022). (in Chinese)
13. H. Zhang. Carbon emission calculation and carbon emission reduction strategy for flash smelting process. Nonferrous Metals (Extractive Metallurgy). 109-112 (2022). (in Chinese)
14. China Nonferrous Metals Industry Association (CNMIA), Data from “The yearbook of nonferrous metals industry of China 2015.”.
15. R. Schmuch, R. Wagner, G. Hörpel, T. Placke, M. Winter, Performance and cost of materials for lithium-based rechargeable automotive batteries. Nature Energy 3, 267-278 (2018).
16. Argonne National Laboratory (ANL), Data from “Update of Bill-of-Materials and Cathode Chemistry addition for Lithium-ion Batteries in GREET2020.”. Available at

<https://greet.es.anl.gov/publication-bom_lib_2020>

1. F. Duffner, et al. Post-lithium-ion battery cell production and its compatibility with lithium-ion cell production infrastructure. Nature Energy. 6: 123-134 (2021).
2. J. B. Dunn, L. Gaines, J. C. Kelly, C. James, K. G. Gallagher, The significance of li-ion batteries in electric vehicle life-cycle energy and emissions and recycling's role in its reduction. Energ. Environ. Sci. 8, 158-168 (2015).
3. H. Hao, Z. Mu, S. Jiang, Z. Liu, F. Zhao, GHG emissions from the production of lithium-ion batteries for electric vehicles in China. Sustainability-Basel 9, 504 (2017).
4. G. Harper, et al., Recycling lithium-ion batteries from electric vehicles. Nature 575, 75-86 (2019).
5. X. Sun, X. Luo, Z. Zhang, F. Meng, J. Yang, Life cycle assessment of lithium nickel cobalt manganese oxide (NCM) batteries for electric passenger vehicles. J. Clean. Prod. 273, 123006 (2020).
6. S. Jiang, et al., Environmental impacts of hydrometallurgical recycling and reusing for manufacturing of lithium-ion traction batteries in China. Sci. Total Environ. 811, 152224 (2022).
